# Ceramides And Stress Signalling Intersect With Autophagic Defects In Neurodegenerative *Drosophila* blue cheese (*bchs)* Mutants

**DOI:** 10.1038/srep15926

**Published:** 2015-12-07

**Authors:** Sarita Hebbar, Ishtapran Sahoo, Artur Matysik, Irene Argudo Garcia, Kathleen Amy Osborne, Cyrus Papan, Federico Torta, Pradeep Narayanaswamy, Xiu Hui Fun, Markus R Wenk, Andrej Shevchenko, Dominik Schwudke, Rachel Kraut

**Affiliations:** 1 National Centre for Biological Sciences; Tata Institute of Fundamental Research; Bangalore, Karnataka, 560065; India; 2 School of Biological Sciences; Nanyang Technological University; Singapore, 637551, Republic of Singapore; 3 Max Planck Institute of Molecular Cell Biology and Genetics, Dresden, Germany; 4 Biotechnology Center (BIOTEC), Technical University of Dresden, Dresden, Germany; 5 ABSciex, Darmstadt, Germany; 6 Research Center Borstel, Borstel, Germany; 7 Department of Biochemistry, National University of Singapore, Singapore

## Abstract

Sphingolipid metabolites are involved in the regulation of autophagy, a degradative recycling process that is required to prevent neuronal degeneration. Drosophila *blue cheese* mutants neurodegenerate due to perturbations in autophagic flux, and consequent accumulation of ubiquitinated aggregates. Here, we demonstrate that *blue cheese* mutant brains exhibit an elevation in total ceramide levels; surprisingly, however, degeneration is ameliorated when the pool of available ceramides is further increased, and exacerbated when ceramide levels are decreased by altering sphingolipid catabolism or blocking *de novo* synthesis. Exogenous ceramide is seen to accumulate in autophagosomes, which are fewer in number and show less efficient clearance in *blue cheese* mutant neurons. Sphingolipid metabolism is also shifted away from salvage toward *de novo* pathways, while pro-growth Akt and MAP pathways are down-regulated, and ER stress is increased. All these defects are reversed under genetic rescue conditions that increase ceramide generation from salvage pathways. This constellation of effects suggests a possible mechanism whereby the observed deficit in a potentially ceramide-releasing autophagic pathway impedes survival signaling and exacerbates neuronal death.

Defective sphingolipid metabolism, in particular misregulation of ceramide production and a failure to catabolize complex sphingolipids, leads to neurodegeneration, often accompanied by increased ceramide levels^1^,^2^. While excessive ceramide production can trigger apoptotic mitogen-activated protein kinase (MAPK)-c-Jun Kinase (JNK) activation^3^,^4^, ceramide metabolites are also positive regulators of autophagy, critical for maintaining neuronal health by ridding the cell of pathogenic aggregated proteins and aged mitochondria^5^,^6^. In non-pathological situations, autophagy provides a source of energy under nutrient stress^7^,^8^,^9^,^10^,^11^. For this reason, it has been proposed that ceramides act as a ‘rheostat’ that can lead to cellular catastrophe if pushed out of balance^12^.

Relatively little is known about causal relationships of ceramide misregulation to neurodegeneration in disease models, but genetic manipulation of sphingolipid metabolism has been shown to influence retinal and muscle degeneration in flies^13^,^14^,^15^. Drosophila *blue cheese* (*bchs*) mutants are neurodegenerative, and Alfy, the human homologue of *bchs*, acts as a scaffolding protein that ferries the aggresome via p62/sequestosome (fly Ref(2)p) with Atg5, Atg12, Atg16L, and Atg8/LC3 toward autophagosomal degradation^16^. Ubiquitin and p62 accumulate progressively in *bchs* mutants^17^,^18^, suggesting that some aspect of autophago/lysosomal degradation is faulty. Larval motor neurons also show increased lysosomal size and altered lysosomal transport in *bchs* mutants^19^,^20^. Bchs contains a conserved BEACH domain, involved in vesicle trafficking^21^. Although no specific function has been assigned to the BEACH domain, the same domain is required in FAN (Factor Activating Neutral-Sphingomyelinase) for its activity upon interaction with TNFα receptor^22^ and production of the signaling molecule ceramide. Based on this information, and because of the precedent of ceramide imbalance in several neurodegenerative situations, we hypothesized that the degeneration in *bchs* animals may be related to ceramides, which might then be associated with its known autophagic defects.

Here, we show by lipidomic analysis that ceramide levels in *bchs* mutant brains are unexpectedly increased. Furthermore, we demonstrate that ceramides are centrally involved in the *bchs* degenerative phenotype, by genetically manipulating ceramide levels, and quantitatively measuring the effect on *bchs* degeneration, autophagic clearance, and Akt, MAPK, and stress signaling. Surprisingly, although *bchs* brains have increased endogenous ceramides, and accumulate exogenous ceramide in autophagosomes, degeneration is rescued by genetic backgrounds that increase ceramide availability; conversely, it is exacerbated by catabolism of ceramide and by drugs that lower *de novo* ceramide production.

In this study, genetic modulation of sphingolipid catabolic pathways that increase ceramide load from salvage (recycling) pathways, not only rescued degeneration, but also improved autophagic clearance, and restored sphingolipid-regulated Akt, MAPK, and stress signaling that were dysregulated in *bchs*. This suggests that ceramides that are recycled through an autophagic pathway act positively on Akt- and MAPK survival pathways, while suppressing ER-stress in the degenerative background, and thereby prevent neuronal demise.

## Results

### Neurodegeneration in *bchs* mutants is modulated by genetically or pharmacologically altering ceramide availability

**Figure 1:**
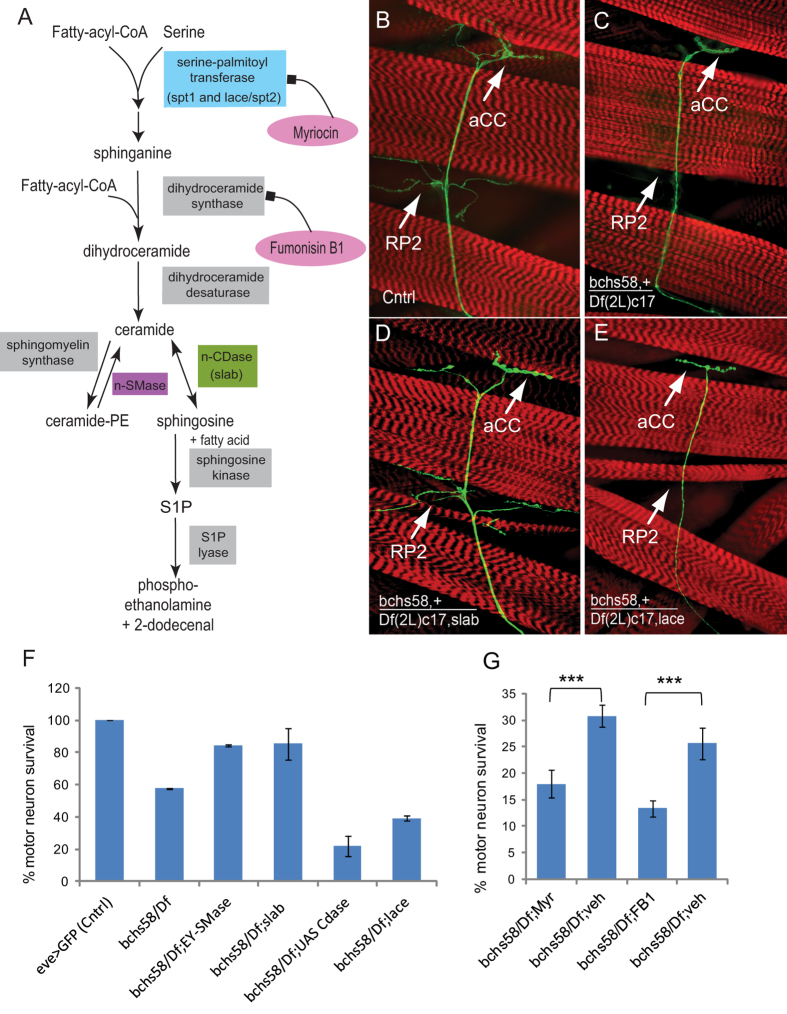
Genetic and pharmacological manipulation of sphingolipid metabolism influences *bchs* neurodegeneration. (**A**) The sphingolipid metabolic pathway showing enzymes that were targeted in colored boxes and their given Drosophila gene names. Pharmacological agents are indicated in pink ovals. (**B–E**) Larval fillets showing motorneuron aCC and RP2 termini expressing mCD8GFP (green) and muscles stained with rhodamine-Phalloidin (red). Loss of motor neurons is evident in *bchs* vs. control (**C**) vs. (**B**). (**D**,**E**) show rescue and exacerbation, respectively, by heterozygosity for *slab*^2^ (**D**) and lace^k05305^ (**E**). (**F**) Quantification of motor neuron loss in *bchs58/Df(2L)c17* and in combination with modifiers. Bars indicate percent survival of RP2 motor neuron in >200 scored hemisegments. For (**F**,**G**) bars represent standard error of the mean. (**G**) Effect on motorneuron survival after feeding wtih 12.4 mM Myriocin (Myr) in vehicle (veh) 50 mM NaOH, or 1 mM Fumonisin (FB1) in water. ***P < 0.001, by Chi-square test.

The potential link of the BEACH domain to sphingomyelin catabolism, perturbations in which can lead to neuronal death^23^,^24^,^25^, led us to ask whether *bchs* degeneration could arise from the mis-regulation of ceramides. If this were the case, we reasoned that *bchs* might be sensitive to genetic changes in sphingolipid biosynthesis or catabolism (Fig. 1A), which in the fly would involve hydrolysis of ceramide (CerPE), the functional equivalent of sphingomyelin. To test this, we used even-skipped Gal4 driving GFP (eve > GFP; Fig. 1B), to label two identified neurons, RP2 and aCC, which in *bchs* null mutants, e.g. *bchs*^58^*/Df(2L)cl7* (denoted *bchs*^58^/*Df*) are lost with a penetrance of ~50%^20^, Fig. 1C,F). Using this assay, different genetic interactors and pharmacological agents could be tested for their effects on motor neuron loss (Fig. 1B–G).

The graph in Fig. 1F summarizes changes in the penetrance of the *bchs* motor neuron phenotype when components of the sphingolipid pathway are manipulated. Reduction of ceramide biosynthesis in the *bchs* null background was first achieved through recombination of *lace*^*k05305*^ (serine palmitoyl transferase 2; *spt2*; CG4162) with the deficiency for *bchs*, *Df(2L)cl7*. Secondly, UAS-nCeramidase (CDase; *slab*; CG1471)^26^, was recombined onto *Df(2L)cl7*, and driven simultaneously with eve > GFP^13^,^27^,^28^. Both these conditions exacerbated motor neuron loss (Fig. 1E,F).

**Figure 2:**
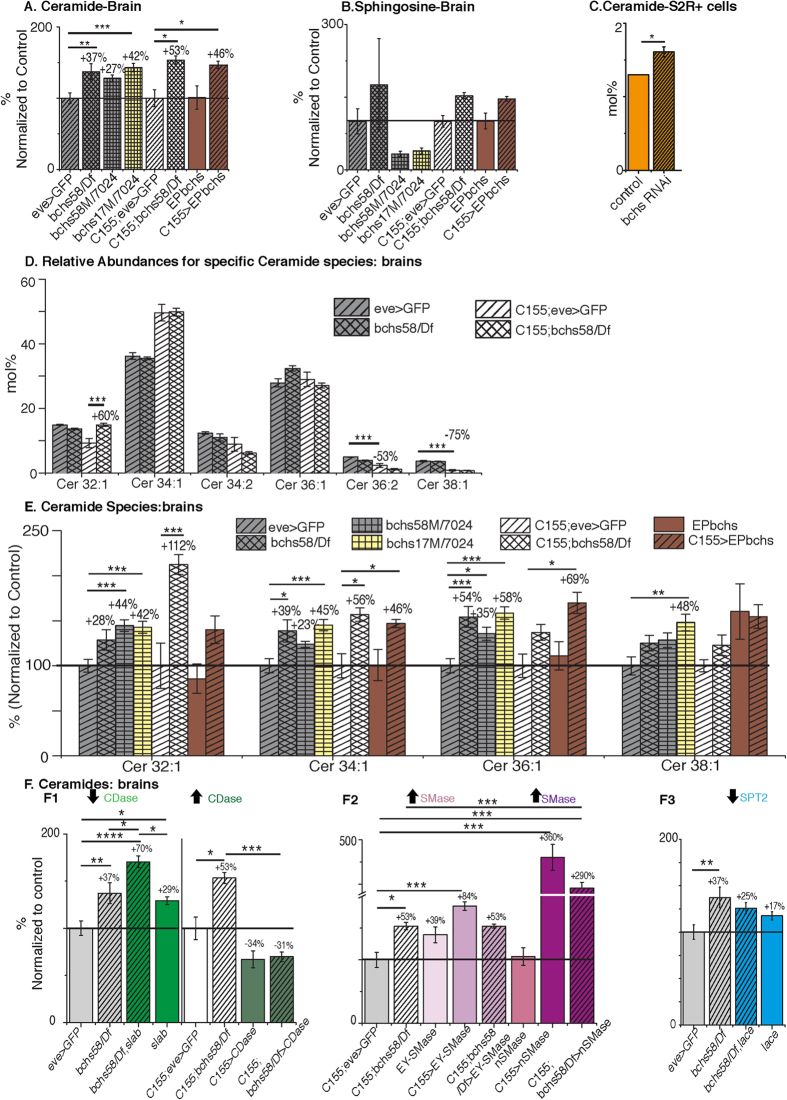
Increased ceramide levels with *bchs* reduction. (**A**) Total ceramide levels are significantly increased in *bchs*^58^*/Df*, *bchs*^17M^*/7024, C155*; *bchs*^58^*/Df* larval brain, and by overexpression in CNS via C155 > EPbchs (EP2299). (**B**) Abundance of C14-sphingosine in larval brain as determined by LC-MS^3^ analysis. (**C**) Ceramide levels in S2R + cells treated with or without (control) *bchs* RNAi, (**D**) Relative distribution profiles for ceramide species in larval brain profiles are similar for *bchs*^58^*/Df* (double hatched bars) in the two genetic backgrounds, with or without C155. Cer 32:1 is changed in *bchs* in the C155; eve > GFP background. (**E**) Major ceramide species in brains compared across the allelic combinations show significant increases in one or more of the major species. (**A**,**B**,**E**) Bars represent mean ± SEM of percent change for lipids (quantified as picomoles/brain) with respect to the genetic controls, eve-Gal4 driving UASmCD8GFP (eve > GFP; grey hatched bars) alone or in combination with C155-Gal4 (C155; eve > GFP; white hatched bars). The control level of 100% is indicated by a horizontal line. (C-D) Bars represent percent molar ratios for total ceramides (normalized to phospholipids) measured from S2R + cells (C) and for ceramide species measured from larval brains (**D**,**F**). Modifiers of *bchs* degeneration change ceramide levels. F1. CDase heterozygote *slab*^2^*/+* increases total ceramides and rescues the degeneration whereas CDase overexpression lowers ceramide levels. F2. nSMase overexpression markedly increases ceramide. F3. *lace*^*k05305*^*/spt2* heterozygotes do not affect ceramide levels. Bars represent mean ± SEM of percent change with respect to genetic control of total ceramide (quantified as picomoles/brain) for manipulations of CDase (*slab*) (1), nSMase (2) and Spt2 (*lace*) (3). Color and hatching schemes represent different *bchs* allelic combinations or *bchs* combined with a given genetic background. Numbers represent percent change relative to the genetic control, set to 100% (indicated by horizontal line). Statistical significance between 2 genotypes is indicated by *p < 0.05, **p < 0.005 and ***p < 0.0005 as determined by ANOVA followed by post-hoc Tukey analyses. Lipidome-wide changes are shown in supplementary sections S1 and S2. Changes in specific ceramide species are summarized in supplementary Figures S3–S5.

Next, an increase in ceramide levels (evidence for which is shown in Fig. 2) in the presence of *bchs* was achieved by introducing heterozygosity for the CDase mutation *slab*^2^ or by driving EY00448 (inserted near the neutral-sphingomyelinase (nSMase) homologue CG12034) in the neurons being scored. Both these backgrounds resulted in rescue of the *bchs* phenotype (Fig. 1D,F), complementing the above findings. None of the modifiers affected neuronal survival significantly by themselves: neuronal survival in *slab*^2^/+ was 98.6%; *lace*^*k05305*^/+ 100%; driving CDase and nSMase (EY00448) resulted in 99.3% and 100% motor neuron survival respectively.

From these genetic interactions, it can be inferred that the neurodegenerative phenotype in *bchs* mutants is highly sensitive to mutations in sphingolipid metabolism that alter ceramide availability (quantitated for the different genetic backgrounds in Fig. 2F). We went on to test whether *bchs* mutants responded in a similar way to pharmacological interference with the sphingolipid biosynthetic pathway (outlined in Fig. 1A) using myriocin, an inhibitor of^29^ and FumonisinB1 (FB1) a fungal inhibitor of dihydroceramide synthase^30^. In agreement with genetic exacerbation by the *spt2(lace*^*k05305*^*)/+* background, which should also reduce *de novo* production of sphingolipids, we found that myriocin exacerbated *bchs* degeneration by 36% and FB1 exacerbated it by 48% (Fig. 1G). Both pharmacological treatments indicate that deficits in *de novo* synthesis of ceramide worsen the neurodegeneration.

### Lipidomics analyses of *bchs* mutants reveal specific changes in ceramides

Since our genetic interaction experiments suggested sensitivity of *bchs* to changes in sphingolipid metabolism, a LC-MS^n^ based lipidomic analysis was performed on brains of *bchs* mutants and interactor genotypes. Lipids were identified based on accurate mass measurements and retention time tags and/or formation of characteristic fragment ions of the sphingoid base^30^,^31^,^32^. Identified lipid classes are listed in Supplementary Table S1. The abundances of lipid species and lipid classes closely overlap with recently reported lipidomic profiles of third-instar larval brain^33^. No significant changes in the major abundant lipids phosphatidylethanolamine (PE), phosphatidylcholine (PC), phosphatidylethanolamine ether (PE-O) and ceramide phosphorylethanolamine (CerPE) were detected across all genotypes (Supplementary Table S2). However, in line with results of our genetic interactions, ceramides, and the long-chain base sphingosine are significantly affected in *bchs* mutants and/or interactors (Fig. 2; Supplementary Table S2).

Unexpectedly in light of its sensitivity to genetic and pharmacological perturbation of ceramides, *bchs* reduction led to *higher* overall ceramides in larval brains and S2R + cell lines (Fig. 2A,C). The two stronger allelic combinations *bchs*^58^*/Df* and *bchs*^17^*/7024* showed an increase of 37% and 42% respectively compared to their genetic background, eve > GFP (Fig. 2A). *bchs*^58^*/Df* and EP2299 also caused increases in ceramides of 53% and 46% in a C155 background (Fig. 2A), without significantly changing sphingosine (Fig. 2B).

Distribution profiles for ceramide species in larval brains were consistent with previous reports for the *Drosophila* lipidome^33^, Cer 34:1 and Cer 36:1 constituting ~70% of total ceramide (Fig. 2D), and the sphingadienes Cer 34:2, Cer 36:2 being one fifth as abundant. It should be noted that the two genetic (control) backgrounds exhibited significant differences for the very low abundant ceramide species- Cer 36:2 and Cer 38:1 (Fig. 2D; white and grey single hatched bars). The main contribution to increased total ceramide levels in bchs mutant brains originated from Cer 32:1, 34:1 and 36:1 (Fig. 2E), but significant changes in all species were seen in at least one *bchs* allelic combination. Increases seen in the sphingadiene ceramides were not significant (Suppl Fig. S3).

### Neurodegeneration in *bchs* is sensitive to changes in metabolic availability of ceramides

Since the genetic interaction studies indicated that *bchs* neurodegeneration may be modulated by ceramide levels, we wanted to find out whether these genetic backgrounds indeed influenced sphingolipid levels in the expected ways.

First, we assessed the effect of the CDase mutant heterozygote with *bchs*^58^*/Df*, which showed a rescue of neurodegeneration (Fig. 1D,F). For *bchs*^58^*/Df, slab*^2^*/+* an even higher, nearly additive increase of 70% in total ceramide was observed as compared to *bchs*^58^/*Df* (37%) and *slab*^2^*/+* (29%) alone (Fig. 2-F1, green). Unlike this rescue, overexpression of CDase in *bchs* (*C155; bchs*^58^*/Df > CDase*) exacerbated the degenerative phenotype, and exhibited a reduction in total ceramide levels by 84% (53% + 31%) compared to *C155*; *bchs*^58^*/Df* alone, similar to the levels seen in CDase overexpression by itself (−34% vs. −31%; Fig. 2-F1, dark green). The genetic formalism that an additive contribution of individual mutants to a phenotype is evidence of parallel mechanisms, suggests that the *additive* increases in ceramides that we see when both *bchs* and one copy of CDase are removed point to parallel (rather than linear) roles of these two genes in a sphingolipid regulatory pathway.

Driving EY-nSMase, which rescued neurodegeneration, increased ceramides as expected, (by 84%) but in the presence of *bchs* this was decreased to the same level ( + 53%) as *bchs* alone (Fig. 2-F2, lavender). Again this is consistent with the genetic formalism of an epistatic relationship, wherein *bchs* may be required for full nSMase activity. UAS-nSMase directly driven by C155 gave a much stronger increase in ceramides, of 360% (Fig. 2-F2, purple). This line induced loss of visible GFP in aCC and RP2 neurons by itself, and was therefore not used for phenotypic interaction studies with *bchs*.

Finally, *lace*^*k05305*^ (*spt2*) heterozygosity, which exacerbated degeneration in *bchs,* reduced *bchs’* ceramide increase to insignificance (Fig. 2-F3). This epistasis of *lace* over *bchs* suggests that *bchs’* increase in ceramides may be partly due to an upregulation of *de novo* ceramide production via *spt*. However, the increases in ceramides in all three genotypes are also likely to be due in part to an upregulation of the salvage pathway from sphingosine^34^. Major ceramide species reflect the same trends that were seen for total ceramides (Supplementary Fig. S4). Ceramide sphingadienes were only significantly changed in the *slab*^2^*/+* backgrounds with or without *bchs* (Supplementary Fig. S5).

We considered that a long chain base-phosphate (LCB-P), (referred to as sphingosine-1-phosphate, or S1P), given its known role as a signaling molecule, in particular with respect to MAPK signaling and autophagy (reviewed in^5^), could be a determinant of neuronal survival in *bchs* mutant neurons. However, the amounts of tissue required to perform the analysis of S1P were prohibitive for larval brain. Therefore, levels of S1Ps as well as sphingosines were measured in 4 day adult male fly heads from *bchs* alone and in combination with *slab*^2^*/+* and CDase-overexpressing backgrounds. (Note that here in accordance with the Drosophila literature, we refer to these species as sphingosine (sph) and S1P, although they contain C14 and C16 bases rather than the mammalian C18 base). No change was seen in S1Ps in *bchs* mutant heads (Supplementary Fig. S6A). Moreover, the analysis shows no obvious correlation between levels of S1Ps or sphingosines with either the genetic rescue or exacerbation effect on neurodegeneration (Supplementary Fig. S6A, B). Therefore, we conclude that sphingosines and S1Ps do not strongly influence *bchs* degeneration.

In summary, the ceramide measurements across all genotypes indicate that manipulations that increase the available ceramide pool act as rescuers of *bchs* degeneration, whereas a reduction of *de novo* synthesis or increased ceramide catabolism exacerbates degeneration. Overall, the metabolic snapshot as assessed by lipidomics supports the findings of the genetic interaction study.

### *bchs* neurons sequester exogenous ceramide in autophagosomes

**Figure 3:**
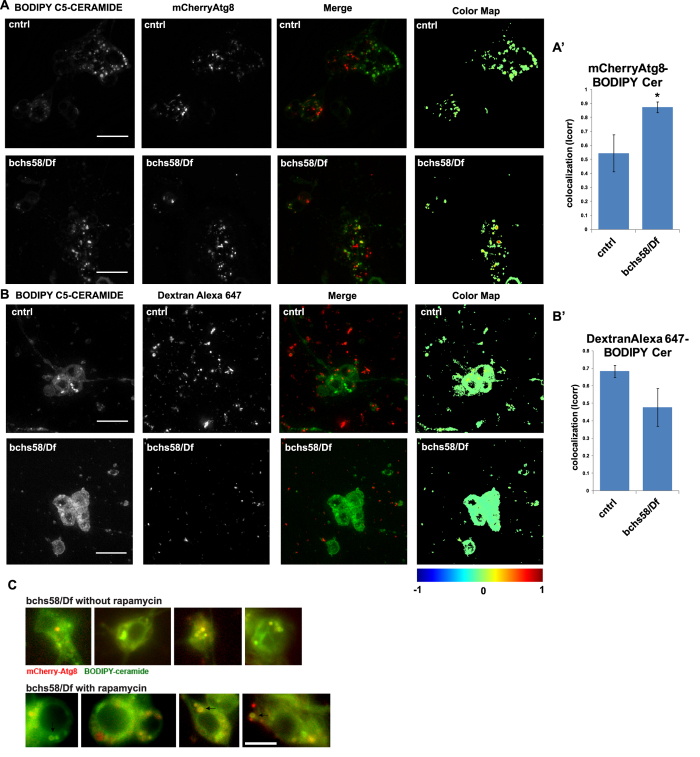
BODIPY-C5 Ceramide in *bchs* primary neurons accumulates aberrantly in mCherry-Atg8-expressing autophagosomal compartments. Live primary neurons incubated with BODIPY C5-ceramide and imaged after chase in label-free medium, in a *bchs*^58^/*Df*; UAS-mCherry-Atg8 expressing background (**A**) and primary neurons from *bchs*^58^/*Df* brains treated with dextran-Alexa647 (**B**) to label endolysosomes. Error bars represent mean ± SEM of correlation coefficient denoting extent of co-localization between ceramide and (**A**) Atg8, or (**B**) Dextran, from four and three experiments, respectively. Scale bar = 10 μm. Co-localization analysis was carried out using the Fiji co-localization color map plug-in (https://sites.google.com/site/colocalizationcolormap/). (**C**) mCherry-Atg8 compartments in *bchs* primary neurons incubated with rapamycin (representative cells in bottom row) appear to clear BODIPY-ceramide more effectively, resulting in hollow compartments rather than the solid BODIPY-ceramide spots seen in untreated neurons (top row).

**Figure 4:**
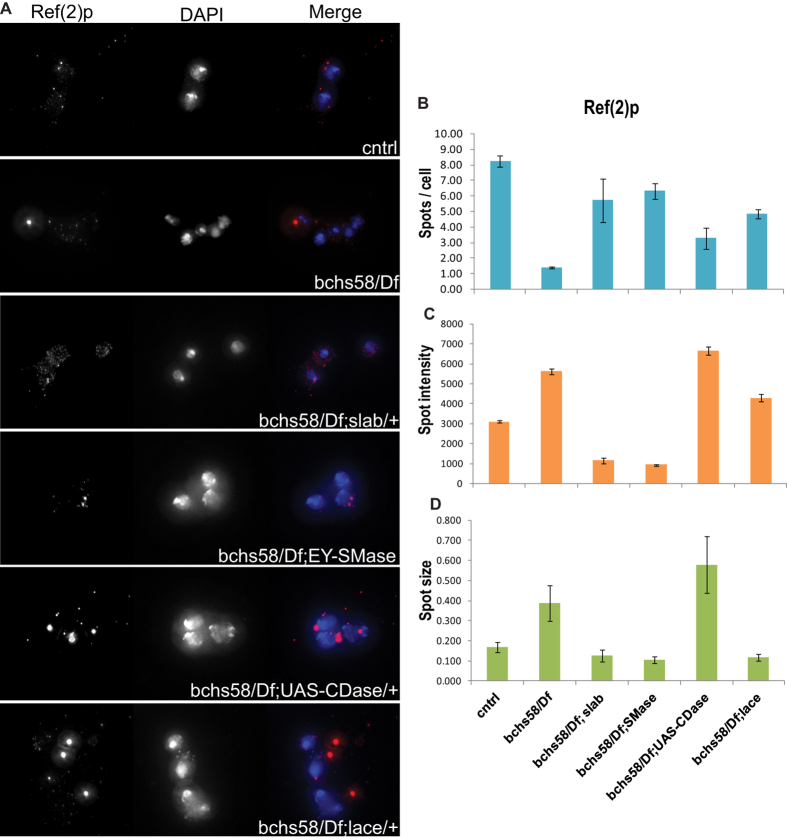
*bchs* and its modifiers affect an autophagic flux marker in primary neurons. Primary neuron cultures of *bchs*^58^*/Df* and *bchs*^58^*/Df* larval brain in sphingolipid modifying genetic backgrounds were immunostained with Ref(2) p antibody (**A**, left panel) and the images were quantified for number of punctae (spots) per cell (**B**), spot intensity (**C**) and spot size (**D**). DAPI was used for nuclear staining. Error bars represent ± SEM.

**Figure 5:**
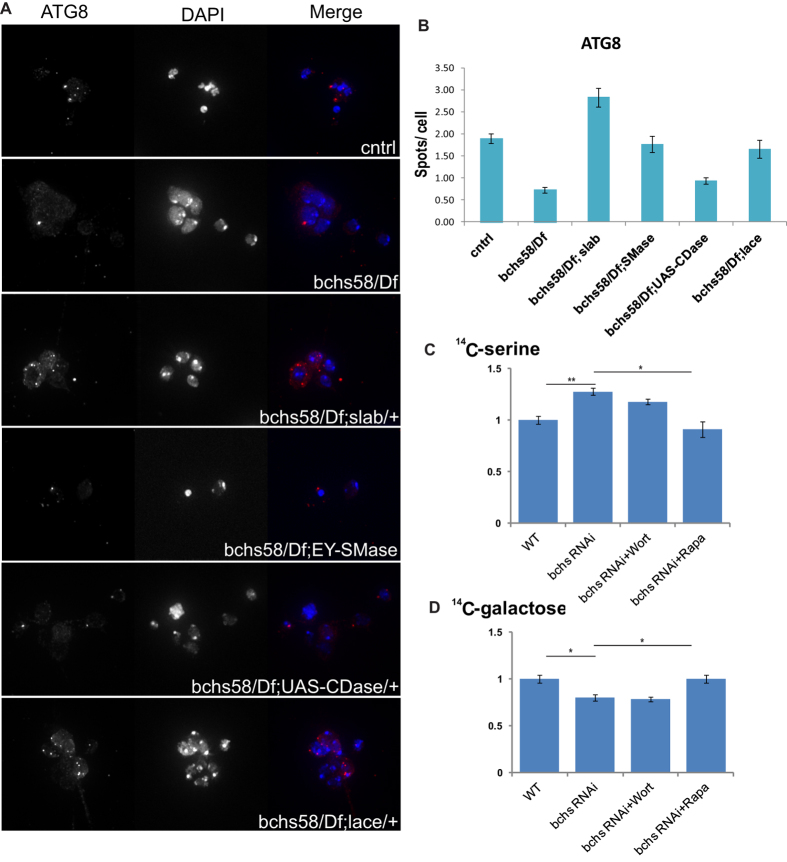
*bchs* mutants alter autophagic induction and imbalance in recycling vs. *de novo* sources of ceramide. Primary neuron cultures of *bchs*^58^*/Df* and *bchs*^58^*/Df* in sphingolipid modifying genetic backgrounds were immunostained with Atg8 antibody (**A**, left panel) and spot number per cell was quantified similarly to Ref(2)p in Fig. 4 (**B**). DAPI was used for nuclear staining. (**C,D**) TLC quantification of total sphingolipids extracted from S2R + cells treated with *bchs* dsRNA and grown in medium containing either ^14^C-serine (**C**) or ^14^C-galactose (**D**) for 24 hrs. For quantification, total lipid was normalized to total protein levels and ^14^C signal was calculated by densitometry of the autoradiograph.

The fact that *bchs* brains accumulated excess ceramides, but were nonetheless rescued by increasing the pool of available ceramides genetically (and conversely were exacerbated by lowering ceramides pharmacologically or catabolically), led us to hypothesize that ceramides, although present in neurons, were perhaps being sequestered in a degradative compartment, unable to be released for signaling or other metabolic activities. Thus, we prepared primary neurons from *bchs* and wild type controls and allowed them to take up exogenous C5-ceramide labeled with fluorescent BODIPY-FL, such that the fate of ceramide could be traced in the cells. This was done in the background of compartmental markers mCherry-Atg8 (labeling autophagosomes^35^ or 10 kDa Dextran–Alexa647, which had been pulse-chased overnight to label late endo/lysosomal compartments^31^,^36^. While wild type neurons displayed relatively little colocalization of BODIPY-ceramide with mCherry-Atg8-labelled autophagosomes, *bchs* neurons had significantly more BODIPY-ceramide in these autophagic compartments (Fig. 3A,A’). The amounts of BODIPY-ceramide trafficking to degradative compartments labeled by Dextran-Alexa647, however, was not changed significantly between *bchs* and wild type (Fig. 3B,B’). This leads us to believe that ceramide, while present overall in higher amounts in neurons, is indeed sequestered and made unavailable in an autophagic recycling pathway that does not function properly (also see figs. 4,5^34^,^37^).

BODIPY-ceramide labelled primary *bchs* mutant neurons were treated with rapamycin in order to determine whether the accumulated lipid would indeed be cleared from autophagosomes more effectively if autophagy were boosted in this way. Figure 3C shows “donut-shaped” mCherry-Atg8-labelled compartments which appear to be cleared of BODIPY-ceramide after rapamycin treatment, whereas the compartments appear solid without the treatment.

### Sphingolipid-modulating genetic backgrounds rescue defects in autophagic clearance in *bchs*

Ceramides have a known role in positively regulating autophagy through activation of stress-associated MAP kinase pathways^11^, and Bchs has been characterized as a pro-autophagic adaptor protein that is required for clearance of aggregated proteins^16^,^38^. We therefore wondered whether the excess ceramides detected in *bchs* brains were associated with an effect on autophagic initiation or clearance. In order to investigate this proposed interaction, we monitored expression of Atg8 and Ref(2)p (the Drosophila p62/sequestosome homolog)^18^ in primary neurons prepared from larval brains of *bchs*, or *bchs* combined with the exacerbating and rescuing sphingolipid-modulating genetic combinations tested in the previous experiments.

Atg8 and p62or/Ref(2)p punctae were analysed using multiple parameters. Analysis of Ref(2)p showed that *bchs* mutants had fewer punctae than wild type controls, but of larger size and higher intensity (Fig. 4A,B–D). Strikingly, these effects were rescued in the backgrounds lacking CDase (*slab*^2^*/+*) and overexpressing nSMase (Fig. 4A,B–D) the same manipulations that rescued neurodegeneration in larvae. In contrast, the exacerbating background UAS-CDase (Fig. 4A,B–D) made the *bchs* changes in intensity and size even more extreme. Combination with *lace*^*k05305*^/*+* (Fig. 4A,B–D) reversed the *bchs* changes partially toward wild type.

Atg8 punctae are shown in Fig. 5A,B quantified as spot number/cell in *bchs* and sphingolipid-altering backgrounds, since other parameters (intensity, size) did not vary significantly. A significant decrease in number of Atg8 punctae in *bchs* primary neurons was rescued back to wild type levels in the same rescuing combinations as were seen for Ref(2)p (*slab*^2^/+ and nSMase overexpression), but remained unchanged in the exacerbating background overexpressing UAS-CDase. Once again, the *lace*^*k05305*^/+ background rescued Atg8 spot number, similar to its effects on Ref(2)p. These results indicate that autophagic flux is perturbed in *bchs* animals, and that this can be strongly ameliorated specifically by enhanced ceramide salvage via an increase in nSMase or reduction of CDase (*slab*). Alternatively, reduction of *de novo* via *lace* appears to have similar rescuing effects on autophagic clearance.

We confirmed that the observed effects on spot number in Atg8, and spot number, size, and intensity in Ref(2)p were pertinent indicators of autophagic flux by treating primary wild type and *bchs*^58^/Df neurons with rapamycin (Supplementary Fig. S7A–C). Notably, rapamycin improved flux in wild type, but not in *bchs*, consistent with our findings that rapamycin does not rescue degeneration of strong *bchs* loss-of-function mutations (submitted elsewhere).

Additionally, no significant effects were seen on Atg8 and Ref(2)p in the CDase, nSMase, and *lace* interacting backgrounds by themselves (Supplementary Fig.7D), suggesting that elevated or reduced ceramide levels in these backgrounds are insufficient to induce changes in autophagic turnover on their own, even though they alleviate the defects in *bchs*.

### Disturbed autophagic flux in *bchs* alters the balance between *de novo* and recycling sources of sphingolipids

Because of the loss of autophagic flux demonstrated in Figs 4 and 5, and the modulation of these phenotypes by genetic changes in salvage (recycling) vs. *de novo* sphingolipid production, we next tested whether the relative amounts of sphingolipids arising from these two pathways might be perturbed in *bchs*, using a method established in mammalian cells^39^. In order to monitor this, S2R + cells were treated with *bchs* RNAi, and *de novo* ceramides were labeled with radioactive ^14^C-serine. A parallel experiment was carried out using ^14^C-galactose, which may be incorporated into glycosphingolipids generated both from *de novo* and from recycled ceramides (i.e. the entire pool of sphingolipids); although it should be noted that glycosphingolipids in mammalian cells are predominantly from recycling sources^40^. Thus, comparing the change in both of these markers should give an indication of any *relative* change in *de novo* vs. total sphingolipids. Bchs knockdown resulted in a significant increase in ^14^C-serine incorporation, compared to a significant decrease in ^14^C-galactose incorporation (Fig. 5C,D), suggesting that there is indeed an increase in *de novo* sphingolipid production vs. that derived from recycling. To test whether this is related to autophagy, we treated the *bchs* RNAi cells with the autophagic inhibitor Wortmannin, or with rapamycin to increase autophagic turnover. Indeed, these cases showed that the *de novo*/salvage ratio could be restored to normal, dependent on autophagy (Fig. 5C,D).

### CDase loss and nSMase overexpression “super-rescue” down-regulated Akt and MAPK pathways in *bchs* brains

To better understand how perturbed ceramide levels in *bchs* mutants might impinge upon survival pathways in neurons, we quantified several key downstream effectors of ceramide signaling in *bchs* larval CNS and adult heads. In light of the role of autophagy in *bchs* neurodegeneration^16^ we tested whether neuronal death may be due to alterations in the mitogen-activated protein kinase kinase MKK4, a sphingolipid-regulated pathway that can induce both autophagy and apoptosis^11^,^41^. Since ceramide also activates PP2A, and thereby negatively regulates Akt, the major convergence point of insulin and TOR growth pathways, we looked for changes in this pathway as well^42^.

**Figure 6:**
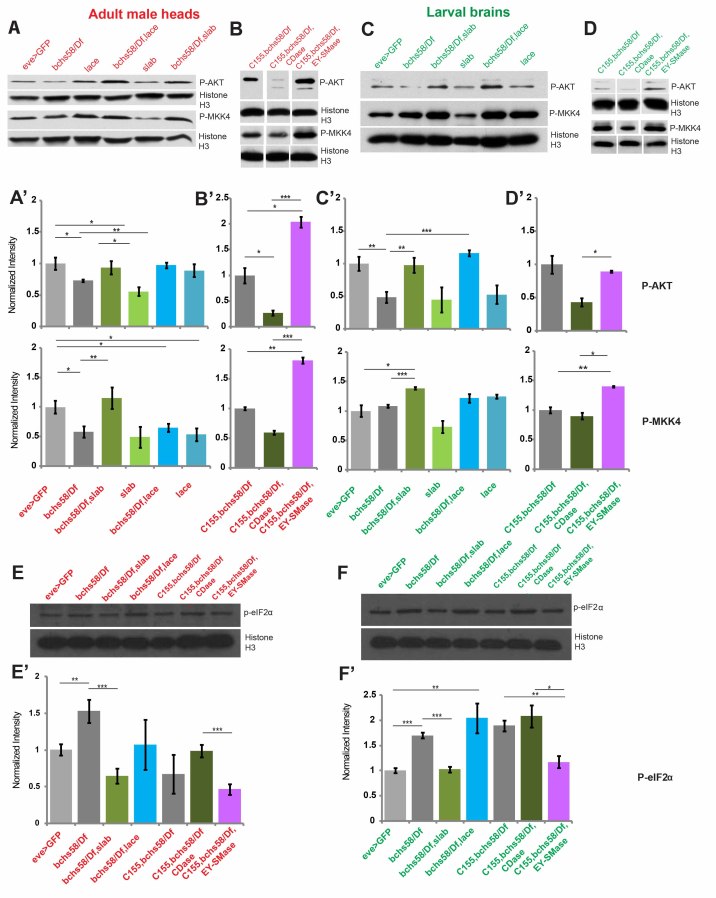
Genetic increases in sphingolipid salvage pathways rescue perturbations seen in ceramide effector pathways in *bchs*. Western blots and quantification of adult heads (**A,A’,B,B’**) and 3^rd^ instar larval brains (**C,C’,D,D’**) from *bchs*^58^/*Df* and *bchs*^58^/*Df* in sphingolipid-modifying genetic backgrounds, probed against p-Akt, p-MKK4, and the ER-stress marker p-eIF2α (**E,E’,F,F’**). For quantification, signals were normalized to histone H3 loading control and analyzed in Image J. Significance was calculated by Student’s-t test (*p < 0.05, **p < 0.01 and ***p < 0.001). Error bars represent ± SEM.

In accordance with increased ceramide levels, *bchs* adult and larval brains (Fig. 6A,A’) have reduced p-Akt levels compared to Akt (total Akt is unchanged across genotypes; Supplementary Fig. S8). This does not seem to be mediated by a drop in PP2A levels (data not shown), although we were not able to measure the more relevant target p-PP2A due to the unavailability of the antibody in Drosophila.

p-MKK4 was surprisingly also reduced in *bchs* adult heads (Fig. 6A,A’), even though increased ceramide would be expected to enhance MLK3 downstream signaling^43^, of which MKK4 is a component. Antibody recognizing Drosophila non-phosphorylated MKK4 was unavailable. Interestingly, reduced p-Akt is also observed in *slab*^2^ heterozygotes, which have elevated ceramides similarly to *bchs* (Fig. 2), while *lace*^*k05305*^ heterozygosity reduces p-MKK4 (Fig. 6A,A’). This observation that genetic manipulations of ceramide levels on their own can affect these signaling pathways, in addition to modulating *bchs’* effects, suggests that it is indeed the changes in ceramides that cause the signaling changes.

Most strikingly, p-Akt and p-MKK4 are restored to levels at or even beyond those found in controls (“super-rescue”) when the rescuing backgrounds *slab*^2^/+ or overexpressing nSMase are combined with *bchs* (Fig. 6A,A’, Supplementary Fig. S8). In contrast, exacerbation by CDase overexpression further reduced p-Akt from its already low levels in *bchs* (Fig. 6B,B’). Thus, these sphingolipid-altering genetic backgrounds’ effects on signaling mirrored their effects on degeneration. The apparently rescuing effect of *lace*^*k05305*^*/+* on p-Akt, however, contrasts with its exacerbation of degeneration.

In order to discover whether ER stress might contribute to (or be a consequence of) the *bchs* phenotype, we also measured p-eIf2α in *bchs* adult and larval CNS^39^. We found p-eIf2α to be consistently elevated in *bchs*, and again rescued by *slab*^2^*/+* and nSMase overexpression. CDase overexpression and *lace*^*k05305*^*/+* by contrast did not modify the *bchs* changes (Fig. 6E,F).

### Bchs knockdown in Drosophila S2 cell line reduces activated Akt and MKK4, but increases stress signals

**Figure 7:**
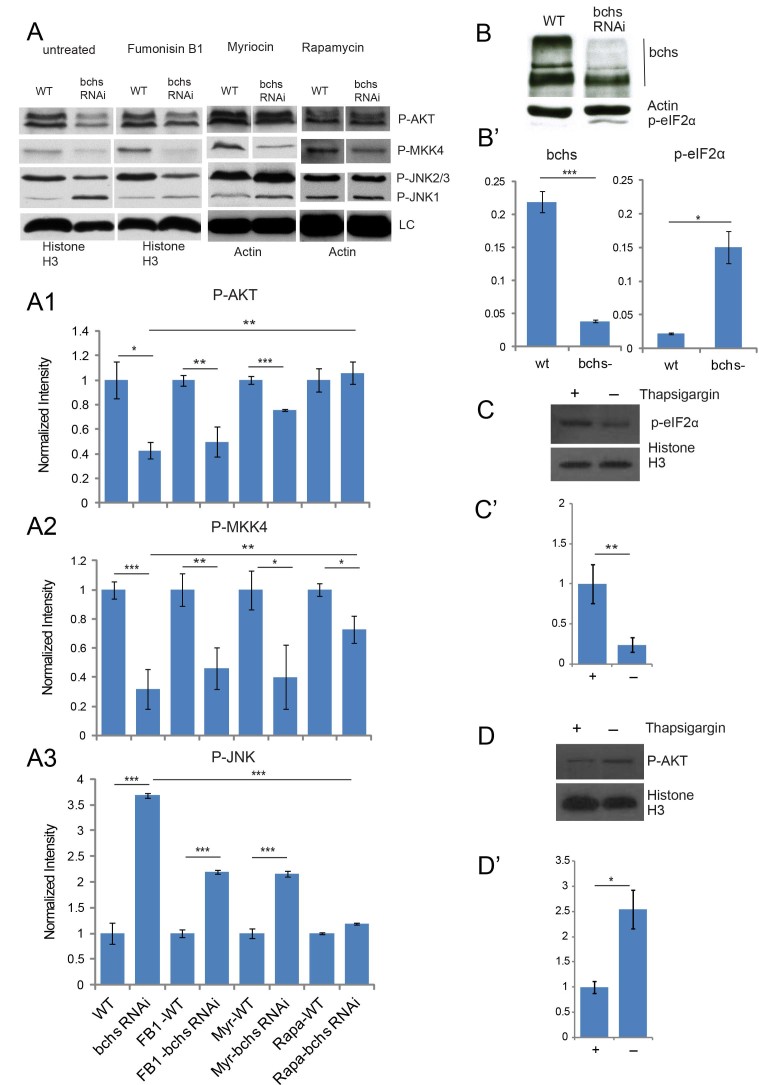
Pharmacological intervention in *de novo* synthesis or autophagy rescues perturbations seen in ceramide effector pathways upon *bchs* knockdown in S2R + cells. Western analysis of S2R + cells after *bchs* knockdown via dsRNA and treatment with Fumonisin B1, myriocin, or rapamycin (**A**) for p-Akt (A1), p-MKK4 (A2), p-JNK (A3) and p-eIF2α (**B,B**’). Knockdown of Bchs product by dsRNA was confirmed in B, B’, and control thapsigargin treatment of S2R + cells and its effect on p-eIF2α and p-Akt are shown in (**C**,**D**). Full blot images of adult heads, larval brains, S2R + cells, and total Akt can be found in suppl Fig. S8. Quantification of blots, shown in bar graphs, was done as in Fig. 6. Error bars represent ± SEM and significance was calculated by Student’s-t test (*p < 0.05, **p < 0.01 and ***p < 0.001).

Although each case in the above Western studies was compared to a control strain of a similar genetic background (i.e. only single chromosomes were exchanged in pairwise comparisons), some genetic variability is difficult to avoid. In order to surmount this intrinsic variability between strains, which we note does affect the quantitative outcome of signaling pathways, we carried out Western analysis of the same markers, Akt, MKK4, and EIf2α, on Drosophila S2R + cells where the Bchs product had been knocked down with dsRNA (Fig. 7A,B). Here, we found similar overall trends in the reduction of survival-associated p-Akt and p-MKK4 (Fig. 7A1), whereas ER stress-associated p-eIF2α was clearly increased in cells (Fig. 7B). As a control, we used thapsigargin treatment of the cells to induce ER stress. As expected, this increased p-eIF2α (Fig. 7C,C’), and lowered p-Akt (Fig. 7D,D’) suggesting that the lower p-Akt seen in *bchs* could also be due to ER stress. Total Akt levels were unchanged across genotypes and treatments (Supplementary Fig. S8); total eIF2α antibody was unavailable.

We next examined whether pharmacological modulation of sphingolipid biosynthesis and/or autophagy using FB1 and myriocin (employed earlier) or rapamycin, also influenced these signaling readouts that were altered in *bchs* brains. There were no changes in these signaling pathways after FB1 and myriocin treatment alone, but these *de novo* synthesis inhibitors did significantly reverse the effects of *bchs* RNAi on p-Akt and p-JNK (Fig. 7A1)—but not MKK4 (Fig. 7A2)—suggesting that Akt and JNK misregulation in *bchs* are mediated at least in part by ceramides generated *de novo*, and that when synthesis is inhibited, these stress-related responses cannot be turned on; however, the stress response may in fact be protective, indicated by increased degeneration under these drugs, and in a *lace* heterozygous background (see Fig. 1).

Interestingly, rapamycin treatment, which increases autophagy^44^ also reversed all the signaling effects of *bchs* RNAi (Fig. 7A1–A3) (and rescued degeneration of a hypomorphic *bchs* mutation; manuscript submitted elsewhere), strongly suggesting that these effects come about as a result of defective autophagy, which has been reported to increase ER stress^45^.

## Discussion

In this report, we demonstrate a decisive role for sphingolipid metabolism in autophagy-related brain degeneration in Drosophila. Even though total ceramide levels are elevated in *bchs* mutants, increasing them further through genetic manipulations rescues both neurodegeneration and survival signaling: neurons appear to survive better in spite (or because) of these higher ceramide levels. Conversely, decreasing ceramide content either by pharmacologically inhibiting synthesis, genetically lowering **de novo** synthesis, or increasing ceramide hydrolysis in a *bchs* deficient background, exacerbates neurodegeneration. This runs counter to the findings of many studies over the last decade documenting increases in ceramide production in degenerative conditions and aging brains^32^,^33^,^46^. Our finding that exogenous ceramide accumulates in autophagosomes, combined with the apparent increase in *de novo* sphingolipid production, provides an explanation for the observed ceramide increases, and suggests that compensatory mechanisms balance the loss of recycled sources of ceramides.

**Figure 8:**
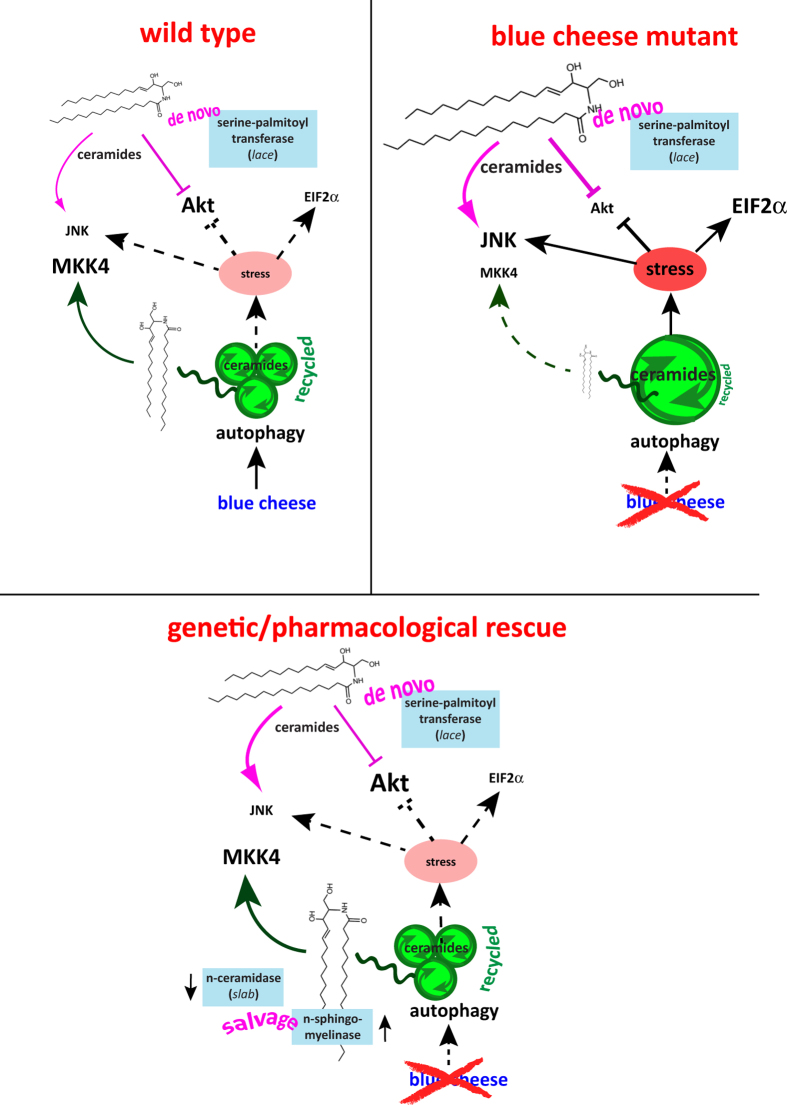
Model summarizing key sphingolipid metabolic pathways contributing to *de novo* (pink) and salvaged ceramide pools (green), whose normal balance (‘wild type’ situation) may be perturbed by *bchs’* blockage of normal autophagic clearance (‘blue cheese mutant’), which increases stress and impinges on survival pathways involving MKK4, JNK, Akt, and EIF2α. Rescue of these signaling pathways, and of neuronal death (‘genetic/pharmacological rescue’), can be achieved by genetic increases in salvage pathways, or by increases in autophagic clearance. Suppression of *de novo* synthesis via spt (*lace*) reduction or treatment with pharmacological agents, only partially rescues signaling pathways (JNK and Akt) but does not rescue neuronal death.

There does not appear to be a one-to-one correlation between total ceramide levels and severity of degeneration, perhaps because ceramides per se can influence a range of cellular outcomes, including growth and/or inflammatory signaling via ERK/MAPK-activation at one end of the spectrum^47^ and JNK-mediated induction of autophagy or apoptosis at the other^5^,^11^. An attractive possible correlate to sphingolipid-related degeneration, suggested by our findings, is that the potentially recyclable pool of ceramide is sequestered in dysfunctional autophagosomes, where it has limited access to signaling cascades mediated by the MAP kinases. Since it appears to be *de novo* ceramide that abnormally suppresses Akt and partially activates JNK in *bchs*, whereas autophagy induction or increases in salvaged ceramide restore all signaling conditions back to normal, we hypothesize that the autophagic defect is the driver of an imbalance in both ceramide sources and overall levels. This triggers stress signals such as JNK and eIf2α, and lowers Akt signaling, which impair neuronal survival. This idea is summarized in the model in Fig. 8.

Exacerbation of degeneration by suppression of *de novo* synthesis, which we found both genetically and pharmacologically, is consistent with the idea that the brain may compensate for inaccessible ceramide pools by increasing *de novo* production. If this is the case, rescue should be seen in backgrounds where less ceramide is broken down to sphingosine, and more is recycled from CerPE by the nSMase homolog, exactly as we observe. Mutants in sphingosine lyase in the mouse show the converse: *de novo* sphingolipid synthesis is down-regulated to compensate for an excess of salvaged ceramides^40^. That this also promotes degeneration implies that the balance of ceramides in the brain from *de novo* synthesis vs. salvage is important for neuronal survival.

Elevated (stored) ceramides may be inaccessible to some signaling pathways in *bchs* brains, since we see a consistent decrease in p-MKK4, even though this MAPK component is thought to be activated by ceramide^48^. p-Akt was also lower, however, as one would expect when ceramides are increased. Notably, both p-MKK4 and p-Akt are restored to normal levels, when the rescuing *slab*^2^ (CDase-) or nSMase backgrounds are introduced. This is presumably associated with the improved autophagic clearance also observed in these backgrounds when combined with *bchs*. Since ceramide is known to trigger JNK and reduce p-Akt by PP2A activation^3^,^49^, increased ceramide levels can cause neurons to die apoptotically. Alternatively, JNK can also phosphorylate Bcl2, leading to release of the beclin/Bcl2 complex, and autophagic initiation^11^. Stress-associated increases in p-JNK signaling may upregulate autophagy, even though this autophagy may be ineffectual, as seen in *bchs*. All of these effects are consistent with phenotypes we observe in *bchs*, including the defective clearance of Ref(2)p/p62-compartments, ceramide storage, increased p-eIF2α and p-JNK stress signaling, and decreased p-Akt. We postulate that when these stress pathways in *bchs* are blocked, e.g. by suppression of *de novo* ceramide synthesis via *spt2* mutation or pharmacological inhibition, this leads to an inadequate response to these stresses while Akt-driven growth signals persist, and these conflicting signals exacerbate neurodegeneration.

The strong rescue of *bchs* neuronal survival and Akt/MAPK signaling by CDase loss may seem paradoxical when both of these genotypes have similar phenotypes (in terms of ceramide increase as well as signaling defects); such genetic interactions, however, are typical for genes that mutually oppose each other, suggesting that Bchs activity may suppress CDase. Consistent with this possibility, creating the opposite genetic situation via CDase overexpression, instead exacerbates degeneration, loss of p-Akt, and ER stress in *bchs*. Bchs’ known autophagic function has been limited to the clearance of aggregated proteins, but since lipids also recycle by autophagy^50^, and BODIPY-ceramide accumulates in autophagosomes in *bchs* neurons, it is conceivable that Bchs has a role in controlling ceramide turnover via CDase.

We note that the excess ceramide generated by overexpression of nSMase in our experiments was damped by loss of *bchs*, indicating that Bchs may be conducive to nSMase generation of ceramide, while it appears to act genetically in opposition to CDase. Thus, by regulating nSMase and/or CDase activity in turn, Bchs may alter the availability of a ceramide pool, and at the same time may encourage autophagic release of recycled sources of ceramide that carries out particular signaling functions, thereby influencing the survival of neurons.

## Materials and Methods

### Fly strains

Animals were raised in standard conditions on Bloomington semi-defined medium (http://flystocks.bio.indiana.edu/Fly_Work/media-recipes/media-recipes.htm). Loss of motor neurons was assayed using the RRa-Gal4(eve-gal4), mCD8GFP line^27^ in mutant alleles as described in^20^,^51^. *bchs* alleles 58M and 17M were generated from precise excision of the EP2299 element in *bchs*^52^ and *bchs*^17^ reported in^51^. Other fly stocks were: *lace*^*k05305*^, UAS-Ceramidase (CG1471)^13^ and *slab*^2^ (CG1471)^13^,^28^, and C155-gal4. nSMase (CG12034) was manipulated using the insertion allele EY00448 (Bloomington Drosophila Stock Center) and a UAS-nSMase line. A detailed description of genotypes used in the lipidomic study is found in Supplementary item S9.

### Generation of UAS-nSMase

Full-length nSMase (CG12034) was amplified from cDNA (DGRC LD24865) using the 5′ primer GGCTCCGCGGCCGCCATGTTGCTGCTGGAGCTGAAC NotI site upstream of ATG and the 3′ primer GGCTCCGGCGCGCCCGTAGAAGTACTCGTACTTCTG AscI site. The product was cloned into Gateway pENTR using the introduced NotI and AscI sites and subcloned into pTW (DGRC #1129) using LR Clonase II (Life Technologies). Transgenic flies were made by BestGene, Inc (California, USA).

### Larval Immunostaining and drug treatments

Larval fillets were immunostained as previously described^20^. Image stacks were acquired on a Deltavision fluorescence microscope (Applied Precision) at 20× (0.75 NA), and projected. Newly hatched larvae were raised in 500 μl of medium containing 10 μl of 12.4 mM Myriocin in 50 mM NaOH or 1 mM Fumonisin B1 in water.

### Western Blotting

Three adult heads or larval brains per lane were crushed in 25 mM Tris pH7.5; TritonX100 0.5%; EDTA 5 mM; NaCl 250 mM with protease inhibitor cocktail and phosSTOP (Roche). Anti-p-SAPK/JNK (Thr183/Tyr185) (CST#9251,1:1000), p-SEK1/MKK4 (Ser257/Thr261) (CST#9156,1:1000), p-Akt (Ser473) (CST#4060,1:2000), Akt (CST#9272,1:1000), β-actin (CST#4967 1:1000), p-eIF2α (Ser 51) and Histone H3 (CST#4499,1:2000) and HRP-coupled goat anti-rabbit (Jackson ImmunoResearch,1:20,000) signals were detected with SuperSignal West Pico Chemiluminescent Substrate (Thermo Scientific, Rockford, Illinois), scanned on an HP Scan jet G4050 and quantified with ImageJ (http://rsbweb.nih.gov/ij), by normalizing first to the loading control, then to the day average of total protein over all genotypes per experiment. Student’s t-test was performed to determine significance.

### Larval brain primary neurons and immunostaining

Four to six 3^rd^ instar larvae were washed thoroughly with 90% ethanol, then de-ionized water; brains were dissected in basal medium M3 + BYPE (without antibiotics/antimycotics, FBS or insulin) freed of imaginal discs, and briefly spun down in 1.5 ml Eppendorf tubes. Medium was replaced with 500 μl of Rinaldi’s saline, then incubated in 200 μl of 0.5 mg/ml collagenase Type I (Sigma) in Rinaldi’s saline for 1 h. 150 μl complete culture medium (M3 + BYPE, 10% FBS [HyClone], 20 μg/ml insulin [from bovine pancreas, Sigma] and antibiotics/antimycotics [Invitrogen]) was added to the brains and triturated ~50 times to dissociate the tissue. Cell suspension was loaded on the centre of the coated coverslip in a 25 mm culture dish, and incubated overnight in a humid chamber. Additional 900 μl of complete medium was then added. Primary neurons were fixed in 4% paraformaldehyde in PBS for 5 minutes at room temperature, washed twice in PBS and blocked in 5% normal goat serum, 0.05% Triton X-100 in PBS for 1 h. Primary antibodies rabbit anti-Atg8 (a generous gift of Katja Kohler) 1:500 and rabbit anti-Ref(2)p, (kindly donated by Ioannis Nezis) 1:1000 in normal goat serum block solution were incubated at 4 °C overnight. Atg8 and Ref(2)p were detected with Cy5 (1:500) and Alexa 488 (1:1500) conjugated rabbit secondary antibodies, respectively. DAPI (1:4000 in PBS) was added for 3 min, and washed in PBS, before mounting on glass slides in 90% glycerol/0.05% propyl gallate.

### Cell labelling with BODIPY C5-Ceramide

5 mg BODIPY C5-ceramide (B-22650; Invitrogen) complexed with BSA in 150 μl of water (0.5 mM stock solution) was stored at −20 °C. 5 μM staining solution was prepared in HBSS/HEPES buffer. Primary neurons were incubated in 10,000 MW Dextran-Alexa647 2 h, washed, and chased overnight with fresh medium. Thereafter cells were rinsed in HBSS/HEPES, incubated with 5 μM BODIPY-ceramide 30 min at 4 °C, rinsed several times with cold medium, incubated in fresh medium at room temperature for up to 15 h, and imaged. Colocalisation analysis was carried out using the Fiji colocalisation color map plug-in (https://sites.google.com/site/colocalizationcolormap/).

### Drosophila S2 cells RNAi, drug treatment

Drosophila S2R + were exposed to dsRNA vs. *bchs* after amplifying *bchs* genomic DNA by PCR using primers (forward) TTAATACGACTCACTATAGGGAGA TGGCGCAAGAACGCAGCTG and (reverse) TTAATACGACTCACTATAGGGAGACTTTTCATGTGTATCCGCTCTG. DNA was purified using QIAquick PCR purification kit and transcribed into dsRNA using T7 Megascript (Invitrogen #AM1334). ~10 μg of RNA was added to each well of a six-well plate and incubated for 3–4 days; knock-down was verified by Western blotting. 15 μM myriocin, 10 μM FB1 or 10 μM rapamycin were added to the RNAi-treated and control cells for 6, 2, or 2 h respectively. For BODIPY-ceramide labelling experiments, cells were incubated in rapamycin continually in the 6 h preceding and during imaging.

### Lipidomic Analyses

#### Larval brain lipidomics

Analyses were carried out on all manipulations used to score motor neuron survival (genotypes listed in Supplementary item S9). Third-instar larval brains were pooled into groups of ten, washed in 20% methanol, freeze-dried, homogenized and extracted with Methyl-tert-butyl ether^53^. Homogenates were spiked with internal standard (Supplementary item, S10) before extraction. LC-MS^n^ analysis was performed on a 1200 micro-LC-system (Agilent Technologies, Waldbronn, Germany) coupled to a hybrid LTQ Orbitrap XL mass spectrometer (Thermo Fisher Scientific, Bremen, Germany) using the TriVersa Nanomate as ion source (Advion BioSciences Ltd, Ithaca NY, US). Lipids were identified based on accurate mass measurements and retention time tags and/or the formation of the characteristic fragment ions of the sphingoid base^54^,^55^,^56^. MS data interpretation was performed with Xcalibur (Thermo Fisher Scientific, Bremen, Germany). Lipid quantities for PE, PC, PE-ethers, CerPE, Cer and Sph were determined by extracting peak areas of high resolution LC-MS^1^ traces with a mass accuracy of ±5 ppm. For Ceramides, Cer 34:1, Cer 36:1, Cer 34:2, Cer 36:2 and C14-sphingosine, MS^3^ was applied to generate specific fragments. Lipids were quantified in picomoles of lipid/brain. Abundance of individual ceramide species is quantified as mol% (picomoles of individual ceramide/picomoles of total ceramide)*100. For a better representation of the MS results related genotypes were normalized to a suitable genetic control. ANOVA and Post-ANOVA pair wise comparisons using Tukey’s test and graphs were computed with OriginPro8.1 (OriginLab Corporation, USA).

#### S2 cell ceramide analysis

Ceramide quantification from S2 cells was analyzed by direct infusion. Samples were infused via nanoflow ESI source (Advion Triversa) into a quadrupole-orbitrap hybrid mass spectrometer (QExactive, Thermo Fisher Scientific). The nanospray source was operated by the Advion Chipsoft software and the spray settings were 0.8 psi and 1.2 kV in both positive and negative mode. Ion transfer tube temperature was set to 200 °C and S-Lens level was set to 50. Full MS spectra were acquired with 140000 resolution (at m/z 400) with an AGC setting of 1 × 10^6^ and a maximum ion injection time of 50 ms. MS/MS-spectra were acquired with a resolution of 70000 (at m/z 400) with AGC setting of 1 × 10^5^ and a maximum ion injection time of 1000 ms. For fragmentation, the precursor isolation window was set to 1 Th centered on each half integer m/z values using a targeted MS/MS (t-MS^2^) method in conjunction with an inclusion list covering the entire mass range between 420–900 in positive mode, so that every mass unit was subject to fragmentation. Normalized collision energy level was set to 20 in positive and 23 in negative mode.

Ceramide and phospholipid species were identified using LipidXplorer by matching the m/z values of the precursor and fragment masses to expected formulas. Ceramides were identified in negative mode by the presence of the a3 and b5 ions as described in^57^ and in positive mode by the long chain base fragments as described in^58^. Phospholipids were identified by the presence of the expected precursor mass and the corresponding fatty acids as described in^52^. The mass error tolerance for the data import was set to 5 ppm and the intensity threshold to 10-times the noise level reported by the Xcalibur software. Quantification of positively identified lipid species was done via normalization to the respective internal standard Cer 18:1/12:0 (20 pmol), PC-OO 36:0 (40 pmol) and PE-OO (50 pmol) which were added upon sample extraction^56^,^59^.

The analysis of LCB and LCB-P in adult head is described in Supplementary item S11.

### Image Analysis

Images of primary neuron cultures were acquired as Z-stacks, with a step of 200 nm in between slices, using the same exposure, light intensity and filter settings for every condition. Collected stacks were then deconvolved in SoftWoRx 4.1.0 (Applied Precision Inc., WA, USA), and converted into 16-bit TIFF stacks in FIJI (http://fiji.sc) using LOCI BioFormats plug-in^60^. Each stack was then Z-projected using the maximum intensity method. Images were inspected under DAPI channel and only single cells, or small aggregates (<5 cells) with clearly separated cell nuclei were used for further spot counting. Because of varying spot intensity relative to cell body, and variable background signal, spots were counted manually, after setting the same display range of values. The cell number N used for calculations varied between conditions (N_average_ ~100), but was not less than N_min_ = 50. The average spot number per cell and the standard error of the mean (SEM) were calculated. To measure spot characteristics, projected images were masked using the Maximum Entropy threshold method^61^. Brightness and size distribution and SEM of selected spots were measured using the Analyze Particles FIJI command.

### Radio-labeling of sphingolipids

Cellular sphingolipids were metabolically labeled according to a published protocol^39^ with [^14^C]-serine (2 μCi/ml) or D1-[^14^C]-galactose (2 μCi/ml). After 24 h, Drosophila S2 cells were harvested and extracted by incubating with 3 ml chloroform/methanol/water (10:5:1, v/v) 24 h at 48 °C. Phospholipids were degraded by alkaline hydrolysis with 100 mM methanolic NaOH, 2 h at 37 °C. Extracts were desalted by reverse-phase chromatography on an RP18 column and neutral lipids were separated from anionic lipids on a DEAE-Sephadex A-25 column, applied to silica gel plates (Merck, Darmstadt, Germany) and run in chloroform/methanol/0.22% CaCl_2_ (60:35:8, v/v) to separate glycolipids or in chloroform/methanol/acetic acid (190:9:1) to separate other sphingolipids. Radioactively labeled sphingolipids were visualized by autoradiography and quantified using ImageJ.

### Preparation of Figures

All figures were assembled in Adobe Illustrator CS5.1 (Adobe Systems) by importing microscopy images (see image analyses) and graphs imported from OriginPro8.1 (OriginLab Corporation, USA) and Microsoft Excel 2011.

## Additional Information

**How to cite this article**: Hebbar, S. *et al.* Ceramides And Stress Signalling Intersect With Autophagic Defects In Neurodegenerative *Drosophila* blue cheese (*bchs*) Mutants. *Sci. Rep.* **5**, 15926; doi: 10.1038/srep15926 (2015).

## Supplementary Material

Supplementary Information
