# Supplementary material for: Ceramides And Stress Signalling Intersect With Autophagic Defects In Neurodegenerative Drosophila blue cheese (bchs) Mutants
Source: Sci Rep. 2015 Dec 7;5:15926. doi: 10.1038/srep15926 (PMC4671070; doi:10.1038/srep15926)
Supplement: Supplementary Information [file srep15926-s1.pdf]

Supporting information for

Ceramides and Stress Signalling Intersect With Autophagic Defects in Neurodegenerative *Drosophila* blue cheese (*bchs)* Mutants

Sarita Hebbar[1,3]#, Ishtapran Sahoo[1,2]#, Artur Matysik[2], Irene Garcia Argudo[2,4], Kathleen Amy Osborne[2], Cyrus Papan[3,5], Federico Torta[7], Pradeep Narayanaswamy[7], Xiu Hui Fun[2], Markus R Wenk[7], Andrej Shevchenko[3], Dominik Schwudke[1,6*] and Rachel Kraut[2,4*]

[1]National Centre for Biological Sciences; Tata Institute of Fundamental Research; Bangalore, Karnataka, 560065; India
[2]School of Biological Sciences; Nanyang Technological University; Singapore, 637551, Republic of Singapore
[3]Max Planck Institute of Cell Biology and Genetics, Dresden, Germany
[4] Biotec, Technical University of Dresden, Dresden, Germany
[5] Sciex, Darmstadt, Germany
[6] Research Center Borstel, Borstel, Germany
[7]Department of Biochemistry, National University of Singapore, Singapore
#These authors contributed equally
*corresponding authors

Contact:
RSKraut@ntu.edu.sg, phone: (65)63162842; fax: (65)67913856
dschwudke@fz-borstel.de, phone: (+49)4537188230, fax: (+49)45371887450

S1, related to Figure 2

Survey of changes in phospholipid species across genotypes. Grey shaded cells indicate no change; red indicates a significant decrease whereas green indicates a significant increase at $p<0.05$ as determined by ANOVA followed by post-hoc Tukey test. Numbers indicate percent change when compared to relevant genetic control.

LIPID SPECIES		Percent change vs. genetic control, eve>GFP							Percent change vs. genetic control, C155;eve>GFP							
		bchs mutants			CDase Perturbation		SPT perturbation		bchs mutants		nSMase perturbation				CDase perturbation	
		bchs 58/Df	bchs 58/7024	bchs 17/7024	slab	bchs 58/Df;slab	bchs 58/Df;lace	lace	C155;bchs 58/Df	C155>EPbchs	C155>EY-nSMase	C155;bchs 58/Df>EY-nSMase	C155>nSMase	C155;bchs 58/Df>nSmase	C155>CDase	C155;bchs 58/Df> CDase
PC	28:0												*192*			
PC	30:0												*107*			
PC	30:1															
PC	30:2	-36	-34	-35	-35			-50					-49			
PC	32:0															
PC	32:1															
PC	32:2															
PC	32:3					42										
PC	34:0															
PC	34:1															
PC	34:2															
PC	34:3				34	59	32	36								
PC	34:4					32										
PC	36:1															
PC	36:2															
PC	36:3				35	70		32								
PC	36:4					48								58		61
PE-O	32:1															
PE-O	34:0					54				160						
PE-O	34:1															
PE-O	34:2															
PE-O	34:3															
PE-O	36:1															

	36:2	35		35	39	48										
	36:3					35										
	38:1									38						
	38:2			36	40	33										
	38:3					34					59	61				60
PE	28:0			-31								78	220			
	28:1			-31	-37			-40								
	30:1															
	30:2							-40								
	32:0															
	32:1															
	32:2															
	32:3	-32														67
	34:0															
	34:1															
	34:2															
	34:3															62
	34:4															
	36:1															
	36:2							-30								
	36:3															
	36:4															
	38:1															
	38:2															
	38:3					45										
CerPE	30:1															
	32:1															
	32:2															
	34:1															
	34:2															
	36:1											53			47	
	36:2															
	38:1					54				78		103				
	38:2															

S2, related to Figure 2

Changes in total levels of lipids belonging to 6 major lipid classes across genotypes. Numbers indicate the percent changes that are significant at $p<0.05$ as determined by ANOVA followed by post-hoc Tukey test. Grey shaded cells indicate no change; red indicates a significant decrease; green indicates a significant increase compared to genetic control.

GENOTYPES			PE	PC	PE-ether	CerPE	Ceramide	Sphingosine
**Percent change as compared to eve>GFP**	bchs mutants	bchs 58/Df					+37%	
		bchs 58/7024						
		bchs 17/7024					+42%	
	CDase perturbation	slab					+29%	
		bchs 58/Df; slab					+70%	
	nSMase perturbation	bchs 58/Df; lace						
		lace						
**Percent change as compared to C155;eve>GFP**	bchs mutants	C155;bchs 58/Df					+53%	
		C155>EPbchs					+46%	
	nSMase perturbation	C155>EY-nSMase					+84%	
		C155;bchs 58/Df>EY-nSMase						
		C155>nSMase					+360%	
		C155;bchs 58/Df>nSMase					+290%	
	CDase perturbation	C155>CDase						
		C155;bchs 58/Df>CDase						+337%

S3, Sphingadienes in *bchs* mutant alleles, related to Figure 2

Diene-ceramides (34:2 and 36:2) are not significantly affected in *bchs* mutant alleles. Cer 34:2 and Cer 36:2 were identified based on LC-Ms^3 generated signature product. Peak heights were used to compute absolute quantities in picomoles/brain unit. For a better representation of the MS results, related genotypes were normalized to a suitable genetic control (fixed at 100% indicated by the horizontal line). Bar graphs represent percent mean ± SEM (with respect to genetic controls) in ceramide levels. The genetic controls include eve-Gal4 driving UASmCD8GFP (eve>GFP) alone or in combination with C155-Gal4 (C155; eve>GFP).

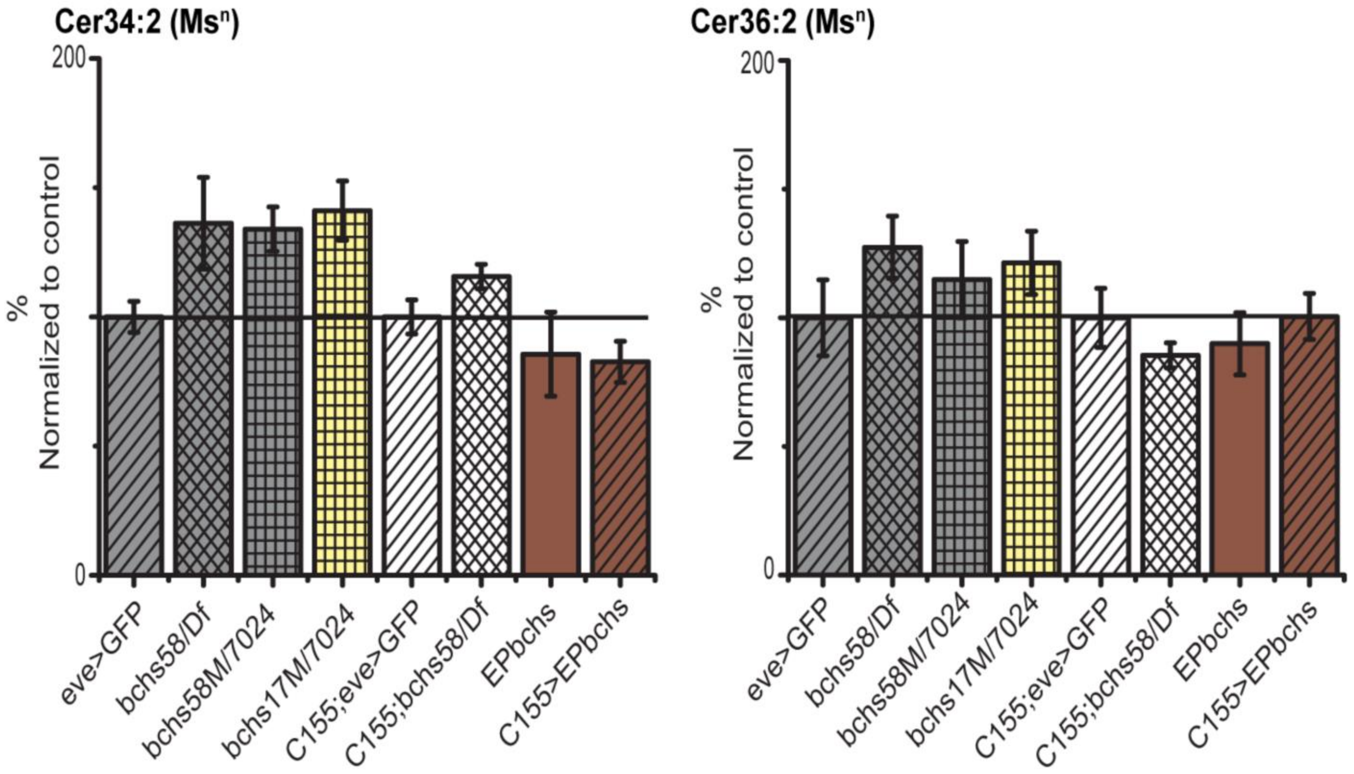

S4, related to Figure 2

Comparison of levels of major ceramide species in *bchs* mutants and interactors. Cer32:1, 34:1 are changed in *bchs* mutants and the modifier combinations. In contrast, Cer 36:1 levels are only changed in combinations that display the highest increases in ceramide levels.

Bar graphs represent mean ± SEM of percent change (with respect to relative genetic controls) for total Cer32:1 (A), Cer34:1(B) and Cer36:1 (C) levels (quantified as picomoles/brain) in manipulations of CDase (green), nSMase (lavender/pink) and *lace*/Spt2 (blue). For a better representation of the mass spectrometric results related genotypes were normalized to a suitable genetic control (fixed at 100% indicated by the horizontal line). The genetic controls include eve-Gal4 driving UASmCD8GFP (eve>GFP; grey hatched bars) alone or in combination with C155-Gal4 (C155; eve>GFP; white hatched bars). Bar colors represent different genetic perturbations, hatched bars indicate combination of multiple genetic perturbations. Numbers represent mean percent change relative to suitable genetic control whose lipid levels are fixed at 100% (indicated by horizontal line). *p<0.05, **p<0.005 and ***p<0.0005 between 2 genotypes indicated by black bar as determined by ANOVA followed by post-hoc Tukey analyses.

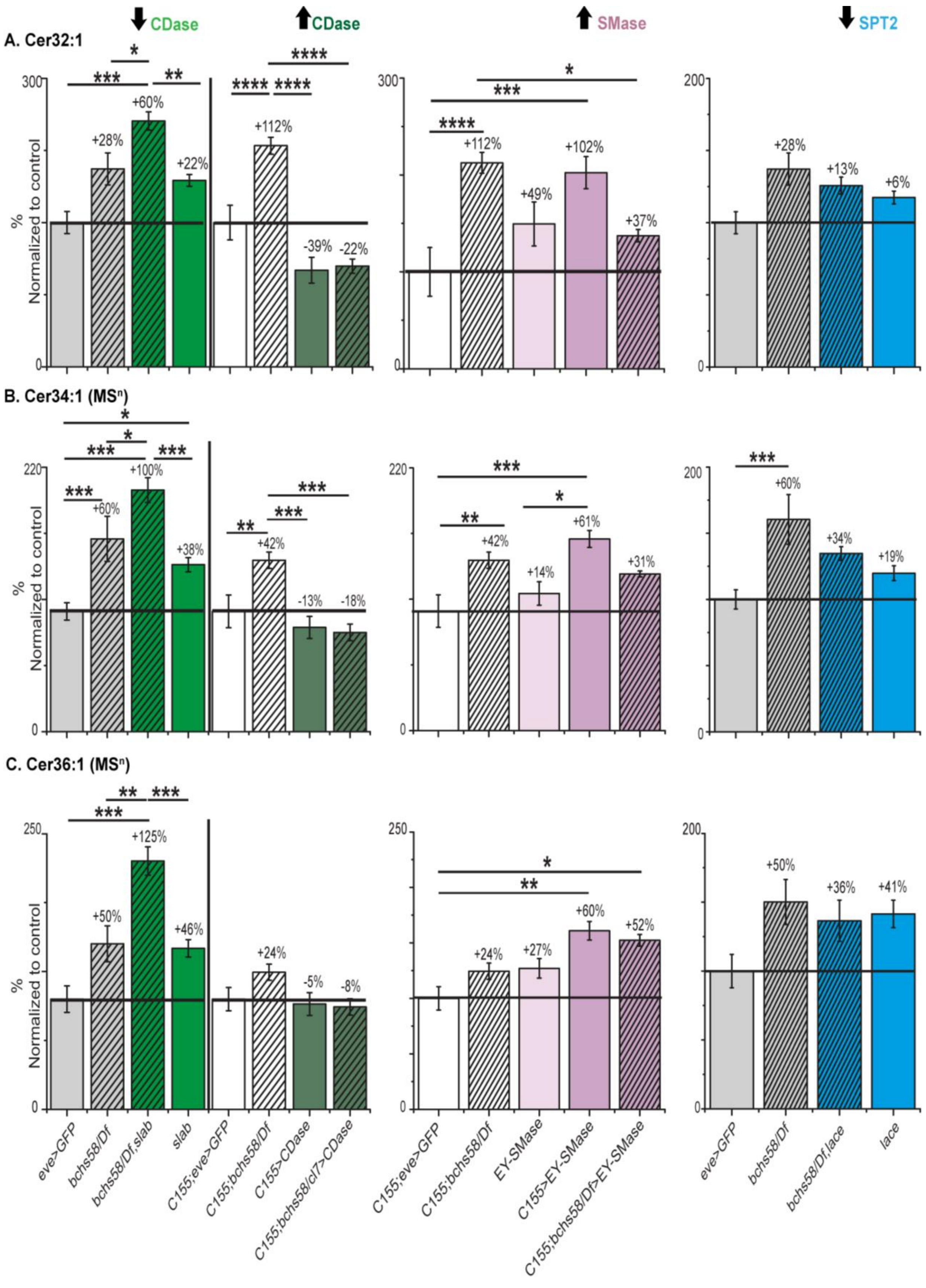

CDase
CDase
SMase
SPT2
A. Cer32:1
B. Cer34:1 (MS^n)
C. Cer36:1 (MS^n)
% Normalized to control
+28%
+60%
+22%
+112%
-39%
-22%
+49%
+102%
+37%
+13%
+6%
+100%
+38%
+42%
-13%
-18%
+14%
+61%
+31%
+34%
+19%
+50%
+125%
+46%
+24%
-5%
-8%
+27%
+52%
+36%
+41%
eve>GFP
bchs58/Df
bchs58/Df,slab
slab
C155;eve>GFP
C155;bchs58/Df
C155>CDase
C155;bchs58/cl7>CDase
EY-SMase
C155>EY-SMase
C155;bchs58/Df>EY-SMase
bchs58/Df,lace
lace

,

S5, related to Figure 2

Trends for diene-ceramides (34:2 and 36:2). Cer 34:2 (A) and Cer 36:2 (B) levels remain unaltered in most genotypes with the exception of *slab* combinations. Bar graphs represent mean ± SEM of percent change (with respect to relative genetic controls) for total Cer34:2 (A) and Cer36:2 (B) levels (quantified as picomoles/brain) in manipulations of CDase, nSMase and *lace/Spt2*. For a better representation of the mass spectrometric results related genotypes were normalized to a suitable genetic control (fixed at 100% indicated by the horizontal line). The genetic controls include eve-Gal4 driving UASmCD8GFP (eve>GFP; grey hatched bars) alone or in combination with C155-Gal4 (C155; eve>GFP; white hatched bars). Bar colors represent different genetic perturbations, hatched bars indicates combination of multiple genetic perturbations. Numbers represent mean percent change relative to suitable genetic control whose lipid levels are fixed at 100% (indicated by horizontal line). *p<0.05, **p<0.005 and ***p<0.0005 between 2 genotypes indicated by black bar as determined by ANOVA followed by post-hoc Tukey analyses.

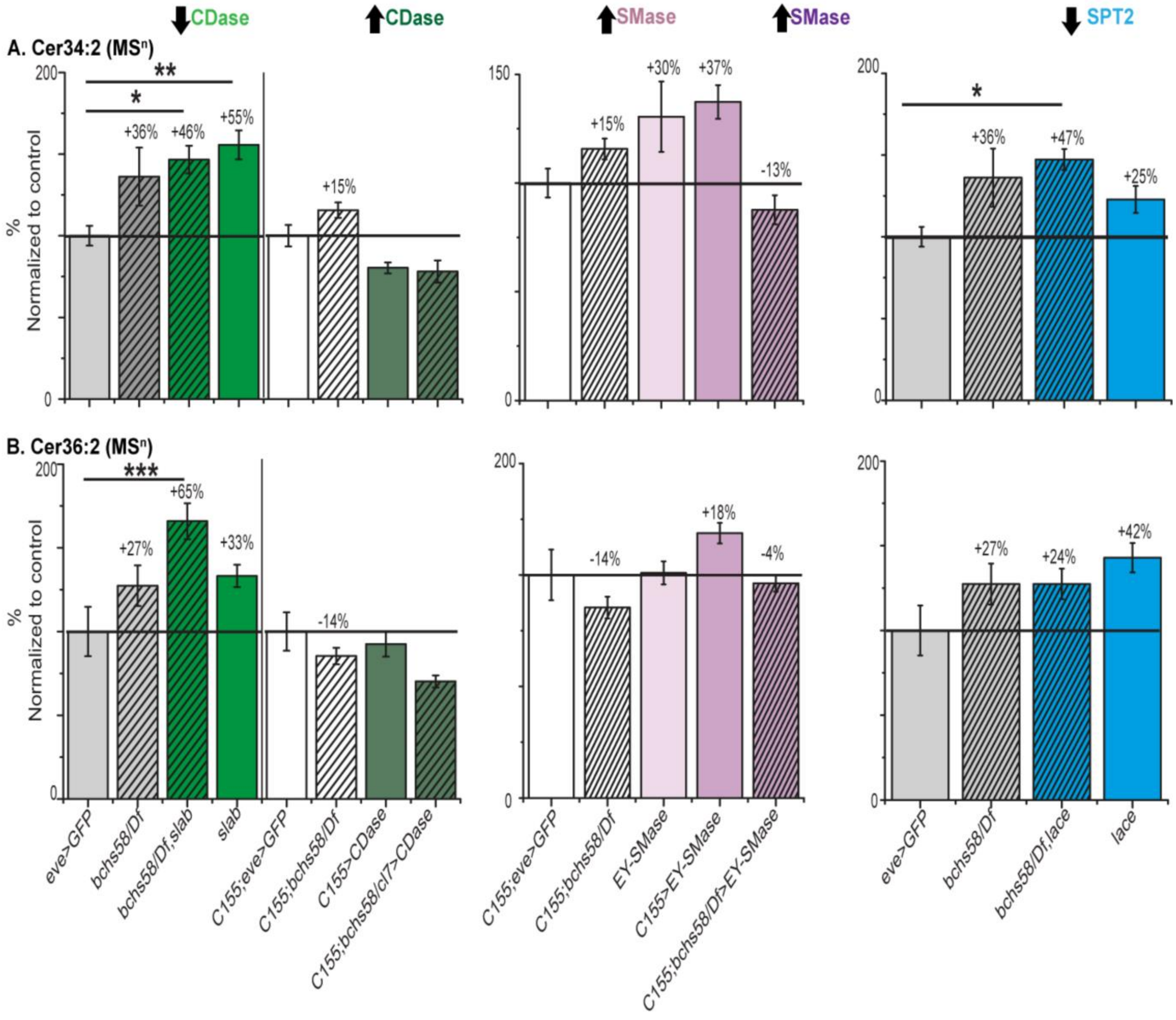
CDase
CDase
SMase
SMase
SPT2
A. Cer34:2 (MSn)
B. Cer36:2 (MSn)
% Normalized to control
**
*
*
***
+36%
+46%
+55%
+15%
+30%
+37%
+15%
-13%
+36%
+47%
+25%
+27%
+65%
+33%
-14%
-14%
+18%
-4%
+27%
+24%
+42%
eve>GFP
bchs58/Df
bchs58/Df,slab
slab
C155;eve>GFP
C155;bchs58/Df
C155>CDase
C155;bchs58/cl7>CDase
C155;eve>GFP
C155;bchs58/Df
EY-SMase
C155>EY-SMase
C155;bchs58/Df>EY-SMase
eve>GFP
bchs58/Df
bchs58/Df,lace
lace

S6, related to Figure 2

Changes in phosphorylated sphingosines (S1Ps) and sphingosines (Sph) do not correlate with exacerbation or rescue of *bchs* mutants in adult head. Comparison of levels of major Long Chain Base-phosphates (LCB-Ps; here referred to as S1Ps) (A) and LCBs (here referred to as sphingosines) (B) species in *bchs* mutant heads alone and in ceramidase (CDase) loss-of-function (*slab*^{2}/+) or overexpressing (OE-CDase) backgrounds showed that the levels of these metabolites do not correlate with rescue or exacerbation of *bchs* phenotypes, as ascertained in other assays. Data represent mean values and standard error of a triplicate experiment. Values are pmol (A) and ng (B) in extracts from 50 adult male 4 day-old heads.

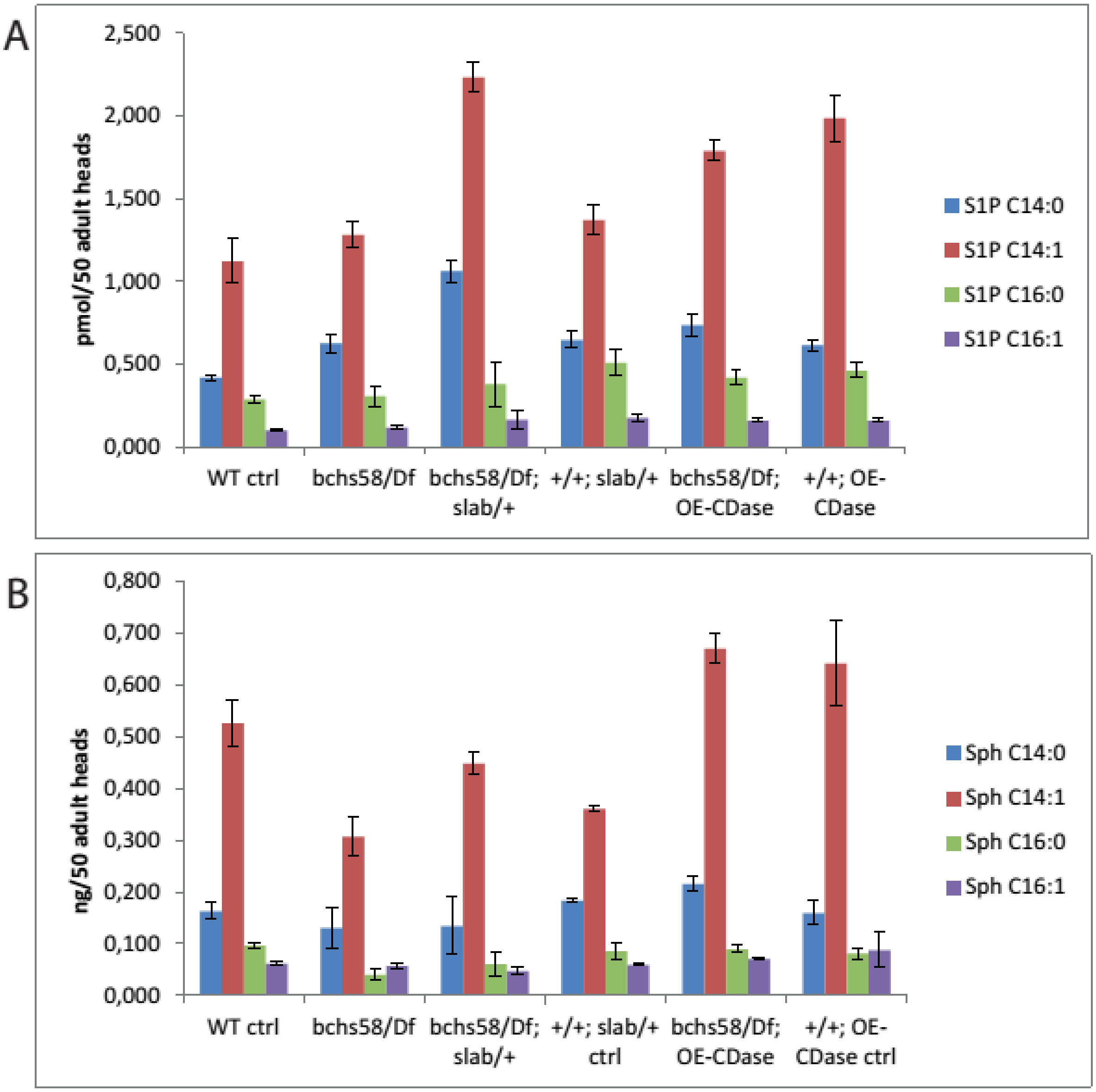

S7, related to Figure 4/5

Effects of rapamycin treatment on Atg8 and Ref(2)p: Atg8 and Ref(2)p were analyzed using various parameters as in fig. 4, to quantitatively assess the changes observed in *bchs* mutant (*bchs*^{58}*/Df(2L)cl7*) and wild type (*w*^{1118}) control (WT) primary cultured neurons under rapamycin treatment. The graphs show the Atg8 and Ref(2)p (A) spot density, (B) mean spot intensity, (C) spot size in the presence (light bars) or absence (dark bars) of rapamycin. (D) Numbers of Atg8 and Ref(2)p spots per cell in the sphingolipid-modifying genetic backgrounds that showed interactions with *bchs* (slab = *slab*^{2}/+; EY-nSMase = *C155; EY-nSMase*/+; UAS-CDase = *C155; UAS-nCDase(slab)*/+; lace = *lace*^{k05305}/+). No significant differences are observed from wild type, given in the graphs in A.

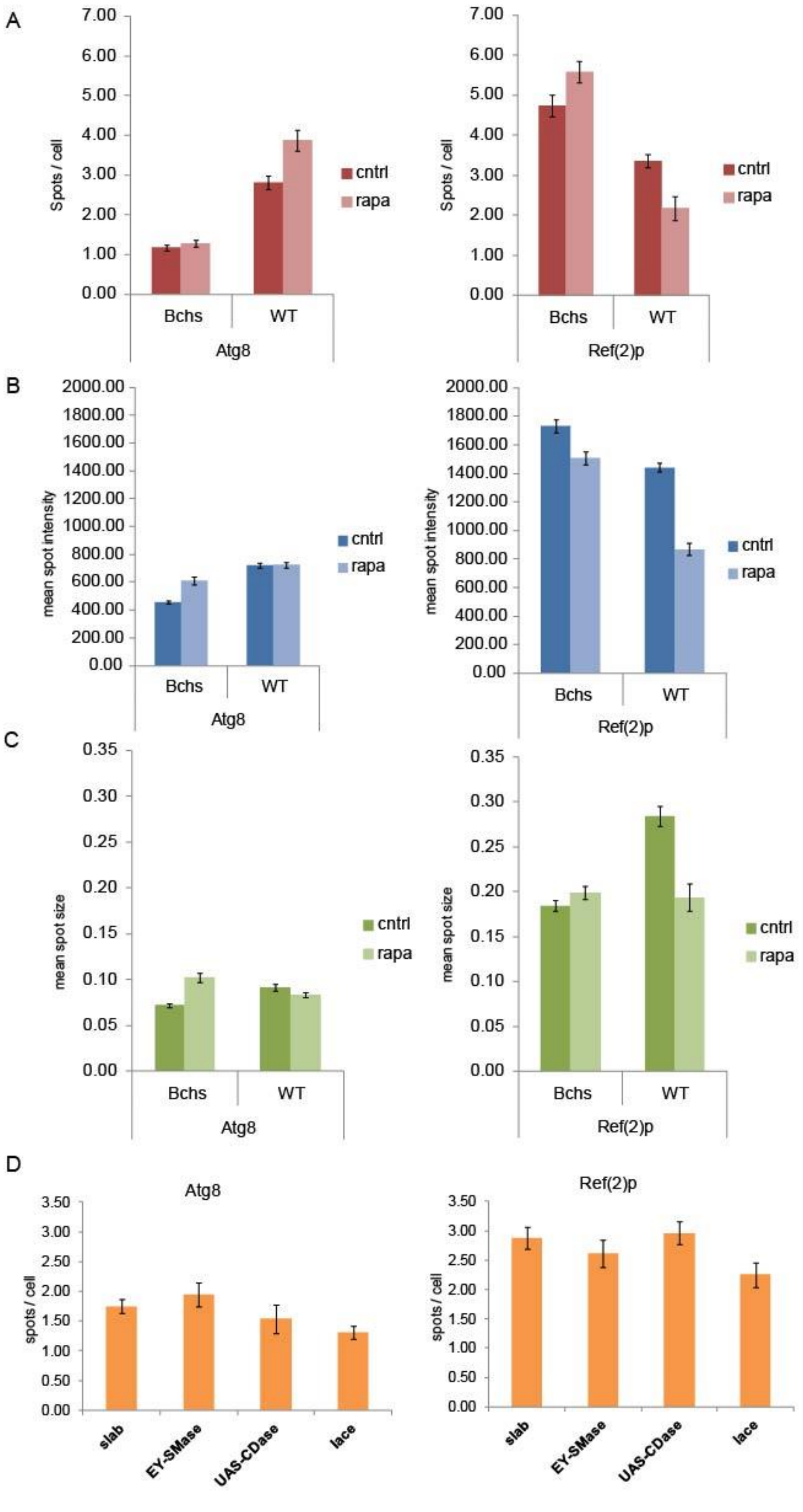
A
Spots / cell
cntrl
rapa
Bchs
WT
Atg8
Ref(2)p
B
mean spot intensity
cntrl
rapa
Bchs
WT
Atg8
Ref(2)p
C
mean spot size
cntrl
rapa
Bchs
WT
Atg8
Ref(2)p
D
Atg8
Ref(2)p
spots / cell
slab
EY-SMase
UAS-CDase
lace

S8, related to Figure 6/7

Full Western blots. Full Western blots of S2R+ cells used in Fig. 7, treated with Fumonisin B1 (A-a), myriocin (A-b) and rapamycin (A-c). Samples were probed against total Akt, p-Akt, p-MKK4 and p-JNK all normalized to histone H3 or actin loading control. Adult head samples (B) and larval brain samples (C) from *bchs* and *bchs* modifiers were probed against p-Akt, p-MKK4 antibodies and the genotypes shown in black bordered boxes were compared and analyzed in Fig 6. Adult head samples and larval brain samples from *bchs* and *bchs* modifiers for total Akt (D) remained unchanged.

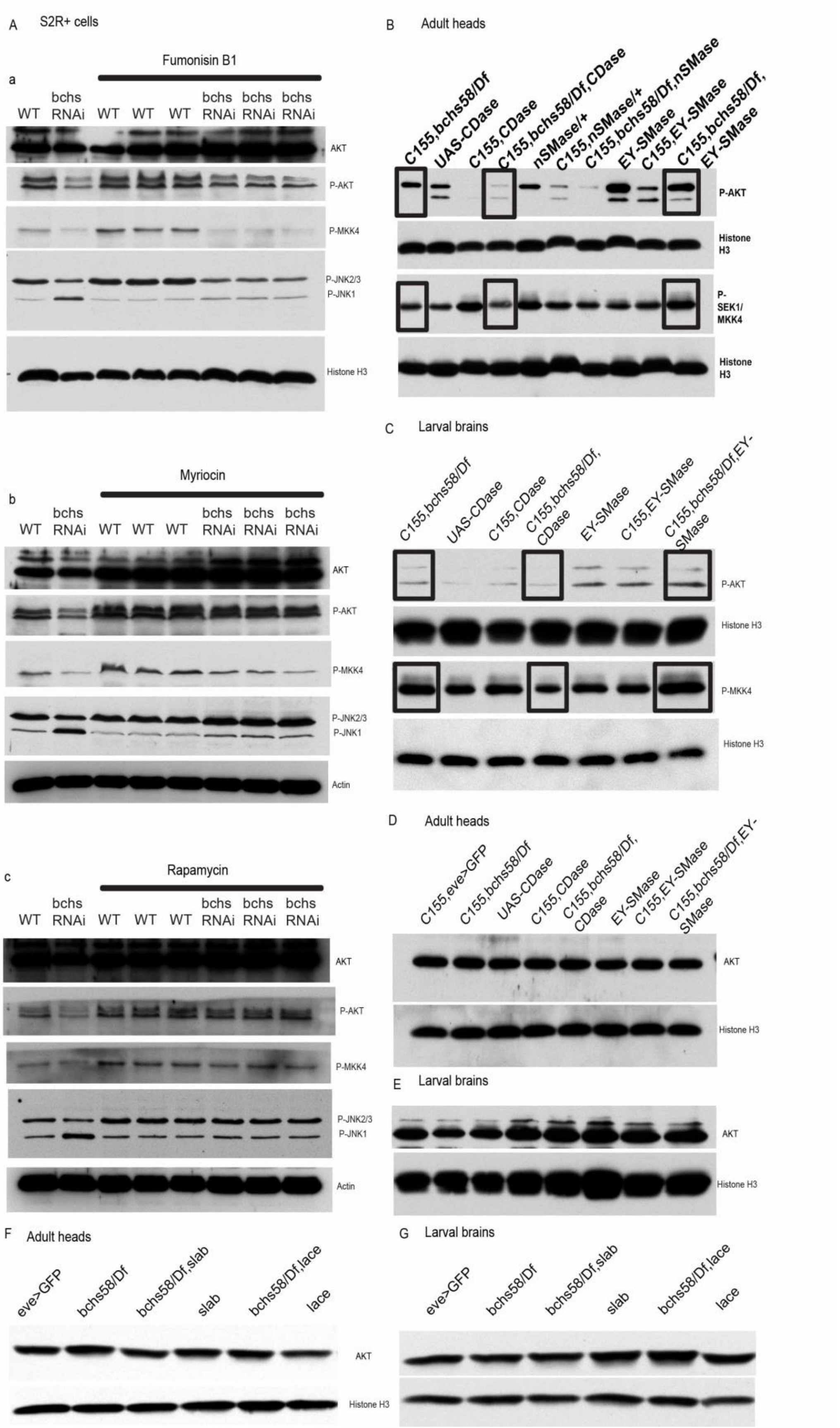
A S2R+ cells
a
Fumonisin B1
WT
bchs RNAi
WT
WT
WT
bchs RNAi
bchs RNAi
bchs RNAi
AKT
P-AKT
P-MKK4
P-JNK2/3
P-JNK1
Histone H3
b
Myriocin
WT
bchs RNAi
WT
WT
WT
bchs RNAi
bchs RNAi
bchs RNAi
AKT
P-AKT
P-MKK4
P-JNK2/3
P-JNK1
Actin
c
Rapamycin
WT
bchs RNAi
WT
WT
WT
bchs RNAi
bchs RNAi
bchs RNAi
AKT
P-AKT
P-MKK4
P-JNK2/3
P-JNK1
Actin
B Adult heads
C155,bchs58/Df
UAS-CDase
C155,CDase
C155,bchs58/Df,CDase
nSMase/+
C155,nSMase/+
C155,bchs58/Df,nSMase
EY-SMase
C155,EY-SMase
C155,bchs58/Df, EY-SMase
P-AKT
Histone H3
P-SEK1/MKK4
Histone H3
C Larval brains
C155,bchs58/Df
UAS-CDase
C155,CDase
C155,bchs58/Df, CDase
EY-SMase
C155,EY-SMase
C155,bchs58/Df,EY-SMase
P-AKT
Histone H3
P-MKK4
Histone H3
D Adult heads
C155,eve>GFP
C155,bchs58/Df
UAS-CDase
C155,CDase
C155,bchs58/Df, CDase
EY-SMase
C155,EY-SMase
C155,bchs58/Df,EY-SMase
AKT
Histone H3
E Larval brains
AKT
Histone H3
F Adult heads
eve>GFP
bchs58/Df
bchs58/Df,slab
slab
bchs58/Df,lace
lace
AKT
Histone H3
G Larval brains
eve>GFP
bchs58/Df
bchs58/Df,slab
slab
bchs58/Df,lace
lace

S9, related to Experimental Procedures

Genotypes used in the lipidomics study.

	GENOTYPE	IDENTIFIER	N*
**Controls**			
Genetic background for mutant	RRa-UASmCD8GFP/RRa-UASmCD8GFP	eve>GFP	5x2
Genetic background for overexpression in nervous system	C155; +; RRa-UASmCD8GFP/+	C155; eve>GFP	3x2
**Genetic perturbation in bchs**			
Null for bchs	bchs58/Df(2)cl7; RRa-mCD8GFP/RRa-mCD8GFP	bchs58/Df	4x2
bchs insertional mutant	EP2299/+;RRa-mCD8GFP/RRa-mCD8GFP	EPbchs	5x2
Null for bchs (without P-element) and with another deficiency	bchs58M/Exel7024; RRa-mCD8GFP/RRa-mCD8GFP	bchs58M/7024	5x2
Loss of function allele	bchs17M/Exel7024; RRa-mCD8GFP/RRa-mCD8GFP	bchs17M/7024	5x2
Overexpression for bchs	C155;EP(2)2299/+; RRa-mCD8GFP/ RRa-mCD8GFP	C155>EPbchs	3x2
Gal-4 in background of bchs null	C155; bchs58/Df(2)cl7; RRa-mCD8GFP/+	C155>bchs58/Df	3x2
**Genetic perturbation in CDase (CG1471) and bchs**			
CDase mutant	slab2, RRa-mCD8GFP/ RRa-mCD8GFP	slab	5x2
bchs mutant + CDase mutant	bchs58/Df(2)cl7; slab2, RRa-mCD8GFP/ RRa-mCD8GFP	bchs58/Df, slab	5x2
CDase overexpression in nervous system	C155;UAS-CDase/+; RRa-mCD8GFP/+	C155>CDase	3x2
bchs mutant + CDase Overexpression in nervous system	C155;bchs 58/Df(2)cl7,UAS-CDase;RRa-mCD8GFP/+	C155;bchs 58/cl7>CDase	3x2
**Genetic perturbation in neutral SMase (CG12304) and bchs**			
genetic background for nSMase overexpression	UAS-nSMase/+;RRa-CD8GFP/+	nSMase	3x2
nSMase overexpression in nervous system	C155; UAS-nSMase/+; RRa-CD8GFP/+	C155>nSMase	2x2
bchs mutant + nSMase Overexpression in nervous system	C155;bchs58/Df(2)cl7,UAS-nSMase; RRa-CD8GFP/+	C155;bchs 58/Df>nSMase	3x2
EY insertion associated with nSMase gene	EY00448,RRa-mCD8GFP/+	EY-nSMase	3x2
C155 driving EY	C155;+:EY00448,RRa-mCD8GFP/+	C155>EY-nSMase	3x2
bchs mutant + C155 driving EY	C155;bchs58/Df(2)cl7, EY00448,RRa-mCD8GFP/+	C155;bchs 58/Df>EY-SMase	3x2
**Genetic perturbation in SPT gene(lace/CG4162) and bchs**			
bchs mutant + lace mutant	bchs58/Df(2)cl7, lace^{k05305}; RRa-mCD8GFP/ RRa-mCD8GFP	bchs58/Df, lace	5x2
CG4162 (lace/SPT) mutant	lace ^{k05305},RRa-mCD8GFP/ RRa-mCD8GFP	lace	5x2

* N represents biological replicates x replicates of mass spectrometric sample acquisition.

S10, related to Experimental Procedures

Internal Standard (IS) mix used

LIPID STANDARD	Catalog No.	SOURCE	Added to extract (pmol)
CerPE-C12 Sphingosyl PE [d17:1]	110753	Avanti Polar Lipids, USA	11.94
PC [17:0-14:1]	LM-1004	Avanti Polar Lipids, USA	6.64
PE [17:1-14:0]	LM1104	Avanti Polar Lipids, USA	6.42
C17-Cer [d18:1/17:0]	860517P	Avanti Polar Lipids, USA	15.61
PE-OO [40:00]	999985P	Avanti Polar Lipids, USA	10.73
Sphingosine [d17:1]	LM-2000	Avanti Polar Lipids, USA	10.64

S11: Adult head LCB and LCB-P analysis

Samples were prepared and analysed as described in Narayanaswamy et al, 2014, with some modifications. 50 heads from adult flies (4 days old) for each genotype were collected in triplicate, flash frozen in liquid nitrogen, and stored at -80 C until lipid extraction.

After addition of LCB and LCB-P (d18:1 LCB-P $^{13}C_2D_2$) standards (4 ng/ml) in 200 µl of butanol:methanol (1:1) the samples were sonicated at room temperature for 30 min. After centrifugation at 14000 g for 10 min the supernatant was split into 2 equivalent aliquots. One aliquot was directly analysed (2 µl injection) by LC-MSMS using a triple quadrupole Agilent 6460 and a 6490 Agilent UHPLC (0.4 ml/min flow) with a C18 Eclipse Plus HHD 2.1 x 50 mm Agilent column. Mobile phase A was 60% Methanol+40% 25 mM ammonium formate and mobile phase B was 90% Isopropanol+10% Methanol 25 mM ammonium formate. The transitions monitored in positive ion mode for LCBs were 246.20/228.2 and 246.2/210.2 (d14:0), 244.20/226.2 and 244.2/28.2 (d14:1), 274.20/256.2 and 274.2/238.2 (d16:0), 272.20/254.2 and 272.2/236.2 (d16:1), 309.20/291.2 and 309.2/273.2 (d18:0 d7 standard).

The second sample aliquot was diluted 10 times in acetonitrile and subjected to LCB-P enrichment on the IMP resin as described in Narayanaswamy 2014. The enriched fractions were dried and reconstituted in 100 µl of methanol. 10 µl of TMS-Diazomethane (2M in hexane) were added and the sample incubated for 20 min at room temperature under gentle mixing. The reaction was stopped by adding 1 µl of acetic acid. The derivatized samples were dried in speedvac and reconstituted in 100 µl of mobile phase before injecting into the chipLC-MSMS Agilent 6490 QQQ system. A customised HILIC-chip containing Amide-80 stationary phase (Tosoh Bioscience, LLC. Montgomeryville, PA, 5 nm particle size, 80 Å pore size) was used for the chromatographic separation (Agilent Technologies Corp., Santa Clara CA). Solvents used for HILIC HPLC: 50% acetonitrile in water containing 25 mM ammonium formate pH 4.6 (solvent A), 95% acetonitrile containing 25 mM ammonium formate pH 4.6. The MRM transitions used for LCB-P measurements in positive ion mode were 410.3/60 and 410.3/113 (d16:0), 408.3/60 and 408.3/113 (d16:1), 382.2/60 and 382.2/113 (d14:0), 380.2/60 and 380.2/113 (d14:1), 440.3/60 and 440.3/113 (d18:1 $^{13}C_2D_2$ standard).

References

Narayanaswamy, P., Sulc, R., Kraut, R., Killeen, K., Grimm, R., Sellergren, B., Torta, F., and Wenk, M.R. (2014). Deep profiling using enhanced analytical workflows reveals new sphingoid base-phosphate species. Analytical Chemistry, 86 (6), pp 3043–3047.
